# A conserved set of maternal genes? Insights from a molluscan transcriptome

**DOI:** 10.1387/ijdb.140121ad

**Published:** 2014

**Authors:** M. MAUREEN LIU, JOHN W. DAVEY, DANIEL J. JACKSON, MARK L. BLAXTER, ANGUS DAVISON

**Affiliations:** 1 School of Life Sciences, University of Nottingham, University Park, Nottingham, UK; 2 Department of Plant Sciences, University of Cambridge, Downing Street, Cambridge, UK; 3 Institute of Evolutionary Biology, University of Edinburgh, Edinburgh, UK; 4 Department of Zoology, University of Cambridge, Downing Street, Cambridge, UK; 5 Courant Research Centre for Geobiology, University of Göttingen, Göttingen, Germany; 6 Edinburgh Genomics, School of Biological Sciences, University of Edinburgh, Edinburgh, UK

**Keywords:** maternal to zygotic transition, mollusk, MBT, MZT, Spiralia

## Abstract

The early animal embryo is entirely reliant on maternal gene products for a ‘jump-start’ that transforms a transcriptionally inactive embryo into a fully functioning zygote. Despite extensive work on model species, it has not been possible to perform a comprehensive comparison of maternally-provisioned transcripts across the Bilateria because of the absence of a suitable dataset from the Lophotrochozoa. As part of an ongoing effort to identify the maternal gene that determines left-right asymmetry in snails, we have generated transcriptome data from 1 to 2-cell and ~32-cell pond snail (*Lymnaea stagnalis*) embryos. Here, we compare these data to maternal transcript datasets from other bilaterian metazoan groups, including representatives of the Ecydysozoa and Deuterostomia. We found that between 5 and 10% of all *L. stagnalis* maternal transcripts (~300-400 genes) are also present in the equivalent arthropod (*Drosophila melanogaster*), nematode (*Caenorhabditis elegans*), urochordate (*Ciona intestinalis*) and chordate (*Homo sapiens, Mus musculus, Danio rerio*) datasets. While the majority of these conserved maternal transcripts (“COMATs”) have housekeeping gene functions, they are a non-random subset of all housekeeping genes, with an overrepresentation of functions associated with nucleotide binding, protein degradation and activities associated with the cell cycle. We conclude that a conserved set of maternal transcripts and their associated functions may be a necessary starting point of early development in the Bilateria. For the wider community interested in discovering conservation of gene expression in early bilaterian development, the list of putative COMATs may be useful resource.

## Introduction

Cell division requires that genome replication and assortment are achieved while cellular function is maintained. In somatic cells, there is continuity of cytoplasm from mother to daughter, so that new nuclei take up the reins of cellular control as transcription of their genomes is resumed after division. In contrast, in the formation of a new organism the early zygote has to perform a similar feat of taking control of a new cell, but the task is made more complex because the gametic pronuclei must be reprogrammed and coordinated before transcription initiation. In animal embryos the zygotic cytoplasm, provisioned by the mother, has been found to contain all the machinery necessary to drive the first stages of embryonic development. This maternal provisioning has been demonstrated through the blocking of transcription from the zygotic genome (Baroux *et al.*, 2008). In transcriptionally-blocked embryos, maternal products are often sufficient to drive the first rounds of cell division, and even the first phases of differentiation (Baroux *et al.*, 2008).

The switch between maternal and zygotic control is called the maternal-zygotic transition (MZT), or the midblastula transition (MBT), and spans the period from fertilisation to the point where maternally provisioned factors are no longer sufficient to deliver normal development (Baroux *et al.*, 2008, Stitzel and Seydoux, 2007, Tadros and Lipshitz, 2009). The MZT is associated with the activation of the zygotic genome. In animal species where fine-scale analyses have been performed, zygotic gene activation has been modelled as two phases (Baroux *et al.*, 2008, Tadros and Lipshitz, 2009). An early phase, involving a few loci, is associated with degradation of maternal proteins and mRNAs, while the second phase is much more extensive and includes genes involved in a wide range of biological processes (Schier, 2007, Tadros and Lipshitz, 2009). Initial, albeit limited, zygotic genome activation has been identified as early as the fertilised zygote (in the paternal pronuclei of mouse, sea urchin and the nematode *Ascaris suum*), and as late as the 256-cell embryo stage (in *Xenopus*) (Baroux *et al.*, 2008, Tadros and Lipshitz, 2009, Wang *et al.*, 2013).

Experimental evidence indicates that the MZT is tightly regulated, and includes the birth of zygotic RNAs and the death of maternal RNAs (Schier, 2007, Stitzel and Seydoux, 2007, Tadros and Lipshitz, 2009), taking place at multiple levels and in a controlled and managed manner. Thus, while many embryos are able to transcribe experimentally introduced DNA, the early embryonic genome is maintained in a state that is incompatible with transcription. Changes in chromatin structure, combined with a dilution of factors such as transcriptional repressors by cell division, allow for the initiation of zygotic transcription. Nonetheless, despite the complexity, it has been suggested that the MZT can be simplified into two interrelated processes: the first whereby a subset of maternal mRNAs and proteins is eliminated, and the second whereby zygotic transcription is initiated (Schier, 2007, Tadros and Lipshitz, 2009).

In zebrafish, maternally-provisioned products from just three genes, *Nanog*, *Pou5f1* and *SoxB1* (known for their roles in embryonic stem cell fate regulation), are sufficient to initiate the zygotic developmental program and to induce clearance of the maternal program by activating the expression of a microRNA (Lee *et al.*, 2013, Leichsenring *et al.*, 2013). In *Xenopus*, increasing nuclear to cytoplasmic ratio is believed to be the controlling element in the switch, with just four factors regulating multiple events during the transition (Collart *et al.*, 2013). However, the generality of these findings remains unknown. Furthermore, while the regulation of RNA transcription (gene expression) has received considerable attention (primarily due to the advances in nucleic acid sequencing technologies), protein expression and turnover rates remain relatively under-studied (Stitzel and Seydoux, 2007). Our knowledge of maternal-to-zygotic transcription phenomena is also largely restricted to the dominant model animal species, with relatively few experimental studies existing for other metazoans.

Although there has been a recent upsurge in interest in the maternal control of embryonic development, especially the MZT (Benoit *et al.*, 2009, De Renzis *et al.*, 2007, Lee *et al.*, 2013, Leichsenring *et al.*, 2013, Tadros and Lipshitz, 2009), the study of maternal factors has played an important part in the history of embryology and development, particularly in the model animal taxa *Drosophila melanogaster* (phylum Arthropoda from superphylum Ecdysozoa), *Caenorhabditis elegans* (Nematoda, Ecdysozoa), *Strongylocentrotus purpuratus* (Echinodermata, Deuterostomia), *Mus musculus, Homo sapiens* and *Danio rerio* (Chordata, Deuterostomia) (Gilbert, 2006). Missing from this roster of models are representatives of “the” superphylum Lophotrochozoa, a morphologically diverse group that includes the Mollusca and Annelida. Two annelid models, *Platynereis dumerilii* and *Capitella telata*, are becoming well established (Dill and Seaver, 2008, Giani *et al.*, 2011, Hui *et al.*, 2009), but model molluscs have been developed for their potential to answer particular questions (e.g. asymmetric distribution of patterning molecules during development; Lambert and Nagy, 2002), or their association with a particular disease (e.g. schistosome transmitting *Biomphalaria*; Knight *et al.*, 2011).

As part of an ongoing effort to identify the maternal gene that determines left-right asymmetry in molluscs (Harada *et al.*, 2004, Kuroda *et al.*, 2009, Liu *et al.*, 2013), we are developing *Lymnaea stagnalis* pond snails as a model because they are one of the few groups that exhibit genetically-tractable, natural variation in their left-right asymmetry, or chirality, and so are ideal systems in which to understand why chirality is normally invariant, yet also pathological when it does vary (Schilthuizen and Davison, 2005). In generating a maternal transcriptomic resource for this species (the chirality-determining gene is maternally expressed; Boycott and Diver, 1923, Sturtevant, 1923), we were surprised to discover that while there are general studies on the composition and regulation of maternal expression (Shen-Orr *et al.*, 2010), there has been no comprehensive description of shared bilaterian maternal genes. One reason may be that no maternal gene resource exists for the Lophotrochozoa, Spiralia or Mollusca. Instead, previous work has described early developmental transcription in the molluscs *Ilyanassa* sp. (Lambert *et al.*, 2010) and *Crepidula fornicata* (Henry *et al.*, 2010), but using combined developmental stage libraries. Here we compare a new 1 to 2-cell *L. stagnalis* transcriptome (presumed maternal) to maternal transcriptomes from selected ecdysozoan and deuterostome species to identify conserved maternally provisioned genes across the Bilateria.

## Results

### *L. stagnalis* embryonic transcriptome sequencing and assembly

Roche 454 sequencing of the two *L. stagnalis* libraries (1 to 2-cell and ~32-cell) generated 192,758 and 218,893 reads respectively, of which 163,004 and 192,552 were 150 bases or longer. The 1 to 2-cell assembly generated more contigs than the ~32-cell assembly, despite having fewer sequences (Table 2). A GC content of 36% for both libraries was approximately the same as previously reported for *L. stagnalis* (Adema *et al.*, 2006, Liu *et al.*, 2013). Merging the two assemblies produced by Newbler and MIRA resulted in fewer, longer contigs. The 1 to 2-cell library generated 11,212 contigs, and the ~32 cell library 9,497 contigs.

### Comparison between maternal transcriptomes

We compared the two developmental transcriptomes of *L. stagnalis* to each other and to six published maternal transcriptomes of roughly comparable depth derived from four deuterostomes and two ecdysozoans (Table 3; Aanes *et al.*, 2011, Azumi *et al.*, 2007, Baugh *et al.*, 2003, De Renzis *et al.*, 2007, Evsikov *et al.*, 2006, Grondahl *et al.*, 2010). For *M. musculus* and *C. elegans*, maternal-only transcripts (present in the oocyte or egg but not in developing embryos) and maternal-zygotic transcripts (found in both oocyte or egg, and after zygotic transcription has started) have been defined. For the mouse, 2,834 genes were maternal-only and 1,796 maternal-zygotic, while for *C. elegans* 2,794 were maternal-only and 2,285 maternal-zygotic (Baugh *et al.*, 2003, Evsikov *et al.*, 2006).

By reciprocal tBLASTx analyses, we identified putatively orthologous genes present in each of the seven species. About one quarter of each of the other maternal transcriptomes, between 900 and 1,900 genes, overlapped with the maternal transcriptome of the pond snail, *L. stagnalis* (Table 4). Surprisingly, 481 of the *L. stagnalis* genes had putative orthologues in all seven taxa (Supplementary Table 1). These 481 orthologues in fact probably represent 439 or fewer distinct genes, as BLASTx analyses revealed that some matched the same sequence in the NCBI nr protein database. This result implies that 5-10% of the maternal transcriptome is conserved and shared across all of the representative taxa (*H. sapiens* 6.1%, *M. musculus* 9.9%, *D. rerio* 10.6%, *C. intestinalis* 11.4%, *D. melanogaster* 7.0%, *C. elegans* 9.0%). We refer to this conserved set as the “conserved maternal transcriptome” (COMAT).

We compared the *L. stagnalis* 1 to 2-cell transcriptome to maternal-only transcripts and maternal-zygotic transcripts from *M. musculus* and *C. elegans* (Baugh *et al.*, 2003, Evsikov *et al.*, 2006) using tBLASTx. The *M. musculus* maternal-only data set matched 1069 *L. stagnalis* transcripts, whereas the *M. musculus* maternal-zygotic data set matched 884 *L. stagnalis* transcripts. Of the 481 COMATs from *L. stagnalis*, 219 were found in the *M. musculus* maternal-only data set and 261 in the *M. musculus* maternal-zygotic data set, indicating a relative over-representation of maternal-zygotic transcripts that are conserved between chordate and mollusc, compared with maternal-only (Fisher’s exact test, 2,834:1,796 maternal-only:maternal-zygotic *M. musculus* versus 1,069:884 maternal-only:maternal-zygotic *L. stagnalis*, *P* < 0.0001), especially when considering COMATs (Fisher’s exact test, 2,834:1,796 versus 219:261, *P* < 0.0001). A similar result was found in comparisons between *L. stagnalis* and *C. elegans* (Fisher’s exact test, 2794:2285 versus 733:929 or 222:259, *P* < 0.0001, *P* < 0.0002). Similar comparisons were also made for maternal transcripts identified as being actively degraded or not degraded in the early embryo (Baugh *et al.*, 2003, Evsikov *et al.*, 2006), but no differences were found.

### Gene ontology analyses

About one-third (31% of the 1 to 2-cell and and 36% of the ~32-cell) *L. stagnalis* transcripts (~3,400 genes) had significant BLASTx matches in the SwissProt database (Table 2). Blast2GO was used to functionally annotate both *L. stagnalis* transcriptomes. Of the 11,212 1 to 2-cell contigs, 4,311 (38%) had a significant BLASTx match, and 3,481 (31%) were assigned GO identifiers. Similarly, of 9,497 ~32-cell contigs, 4,255 (45%) had a significant BLASTx match, and 3,425 (36%) were assigned GO identifiers. For the COMAT subset, all but one of the 481 sequences had a significant BLASTx match, and 435 (90%) were assigned GO identifiers (Supplementary Table 1).

**Fig. 1:**
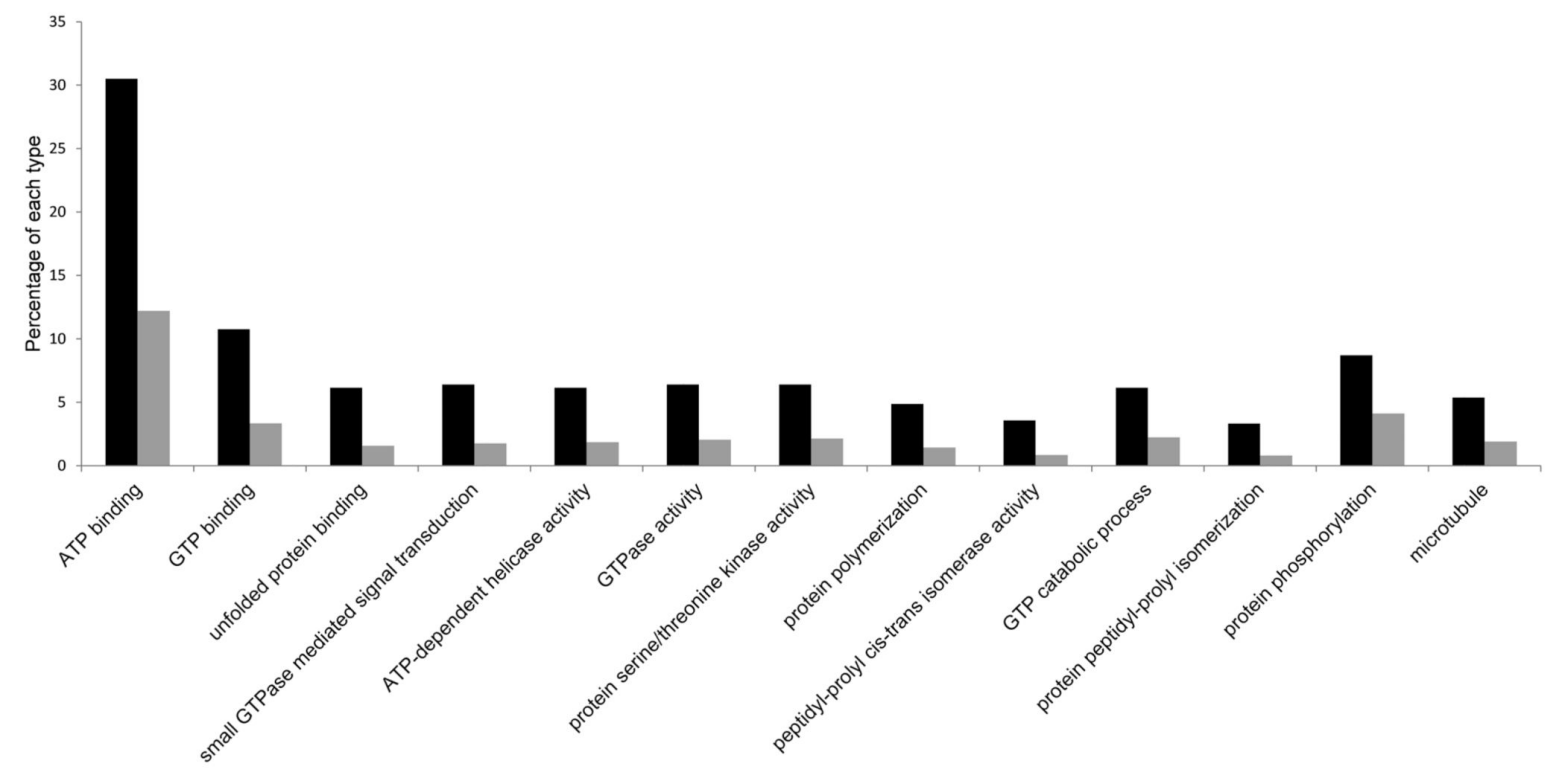
Enrichment of Gene Ontology terms in the conserved maternal transcript (COMAT) subset Highest level GO terms that show the greatest enrichment in COMAT compared with the L. stagnalis 1 to 2-cell transcriptome. Only those comparisons with P < 1E-5 are shown. Black shading: percentage of each type in COMAT. Grey shading: percentage of each type in the 1 to 2-cell transcriptome.

**Fig. 2:**
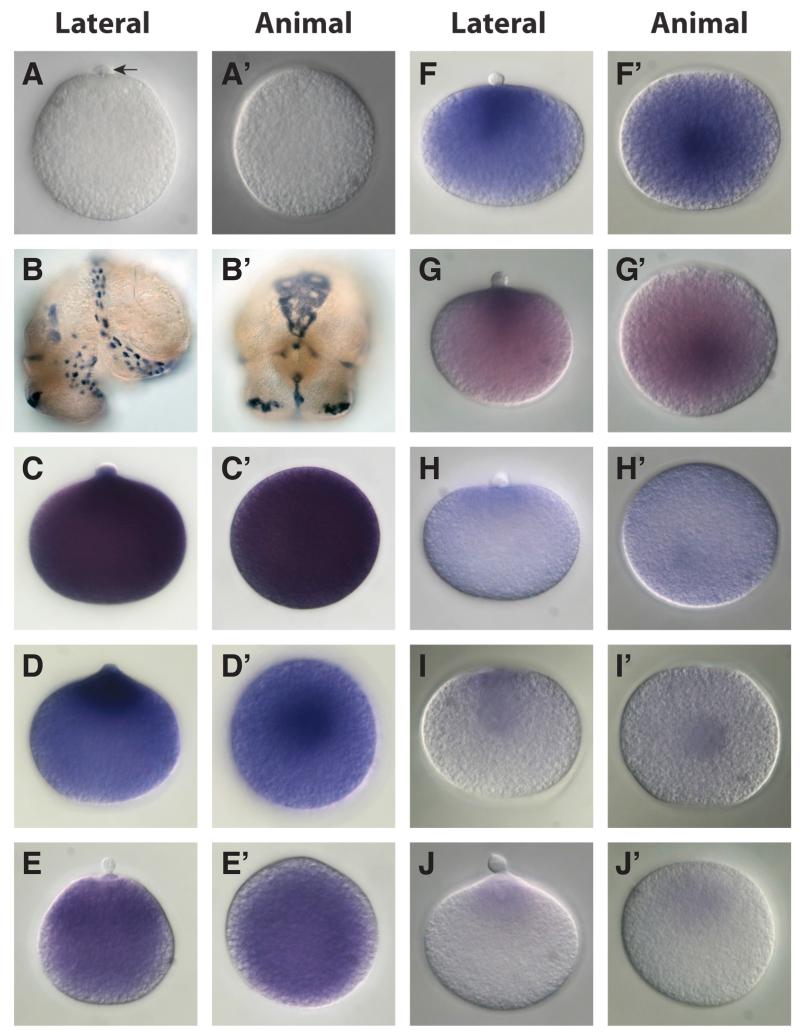
Visualisation of maternal gene product spatial distribution in uncleaved zygotes of *Lymnaea stagnalis* by whole mount *in situ* hybridisation Eight maternal gene products were visualised in uncleaved zygotes relative to a negative control (β*-tubulin).* **(A)** β-tubulin is not detectable in uncleaved zygotes. A polar body is indicated by the horizontal arrow. **(B)** β-tubulin is clearly expressed in ciliated cells of older veliger larvae. **(C)** contig_2724: ATP-dependent RNA helicase dhx8. **(D)** contig_453: heat shock 70 kda protein cognate 4. **(E)** contig_7974: ADP-ribosylation factor 4. **(F)** contig_9053: proteasome alpha 6 subunit. **(G)** contig_579: ergic and golgi 2. **(H)** contig_9016: eukaryotic translation initiation factor 3 subunit i. **(I)** contig_8075: eukaryotic translation elongation factor. **(J)** contig_8318: 78 kda glucose-regulated protein.

The distribution of GO annotations into functional categories revealed no obvious qualitative differences between the 1 to 2-cell and ~32 cell *L. stagnalis* transcriptomes (Supplementary Figure 1). A Fisher’s exact test, with multiple correction for false discovery rate, confirmed that no functional categories were significantly under or overrepresented between the two libraries. In comparison, the COMAT subset was enriched for many functional categories compared with the complete *L. stagnalis* 1 to 2-cell transcriptome (Fig. 1; Table 5; Supplementary Table 2). In particular, GO terms associated with nucleotide metabolism and binding in general were overrepresented in the COMAT subset (Figure 1; Table 5; Supplementary Table 2). The maternal expression of a selected set of the COMAT genes was validated in one-cell zygotes using *in situ* methods (Fig. 2).

### Comparison with human housekeeping genes

The COMAT subset was compared to 3802 well-characterised human housekeeping genes (Eisenberg and Levanon, 2013). All but 38 of the 481 COMAT transcripts had a significant match to this set (92%), indicating that the majority are housekeeping in function, at least in humans. In comparison, of the 4,311 *L. stagnalis* 1 to 2-cell transcripts that had a significant BLASTx match in the NCBI nr protein database, only 2,165 (50%) also had matches to the human housekeeping gene dataset. The conserved maternal gene dataset is therefore highly enriched for putative housekeeping genes (Fisher’s exact test, 2156:4311 versus 443:481, *P* < 0.0001).

We wished to understand if a particular subset of housekeeping genes are over-represented in the COMAT subset, or whether the genes are a random subset of all housekeeping genes. We therefore compared the GO annotations of the 3,802 human housekeeping genes against the subset of 300 human housekeeping genes (Table 6) that were found in the COMAT (a proportion of the COMATs hit the same human gene, hence fewer genes than expected). Similar GO annotations were enriched in this selected pairwise comparison compared with the COMAT as a whole (Supplementary Tables 3 and 4). At the highest level, the same first seven Molecular Functions were found in both *H. sapiens* housekeeping versus *H. sapiens* COMAT, and *L. stagnalis* 1 to 2-cell transcriptome versus *L. stagnalis* COMAT comparisons, with *P* < 5E^−8^ (Supplementary Table 4; ATP binding, GTPase activity, unfolded protein binding, protein serine/threonine kinase activity, GTP binding, threonine-type endopeptidase activity, and ATP-dependent RNA helicase activity). Similarly, the first seven terms relating to Biological Process were also found (*P* < 5E^−8^; anaphase-promoting complex-dependent proteasomal ubiquitin-dependent protein catabolic process, protein polyubiquitination, negative regulation of ubiquitin-protein ligase activity involved in mitotic cell cycle, DNA damage response, signal transduction by p53 class mediator resulting in cell cycle arrest, positive regulation of ubiquitin-protein ligase activity involved in mitotic cell cycle, antigen processing and presentation of exogenous peptide antigen via MHC class I, and TAP-dependent, GTP catabolic process). Thus, the overall conclusion is that the COMAT generally consists of housekeeping genes, but is particularly enriched for a particular subset, including those involved in nucleotide binding functions, protein degradation and activities associated with the cell cycle.

**Fig. 3:**
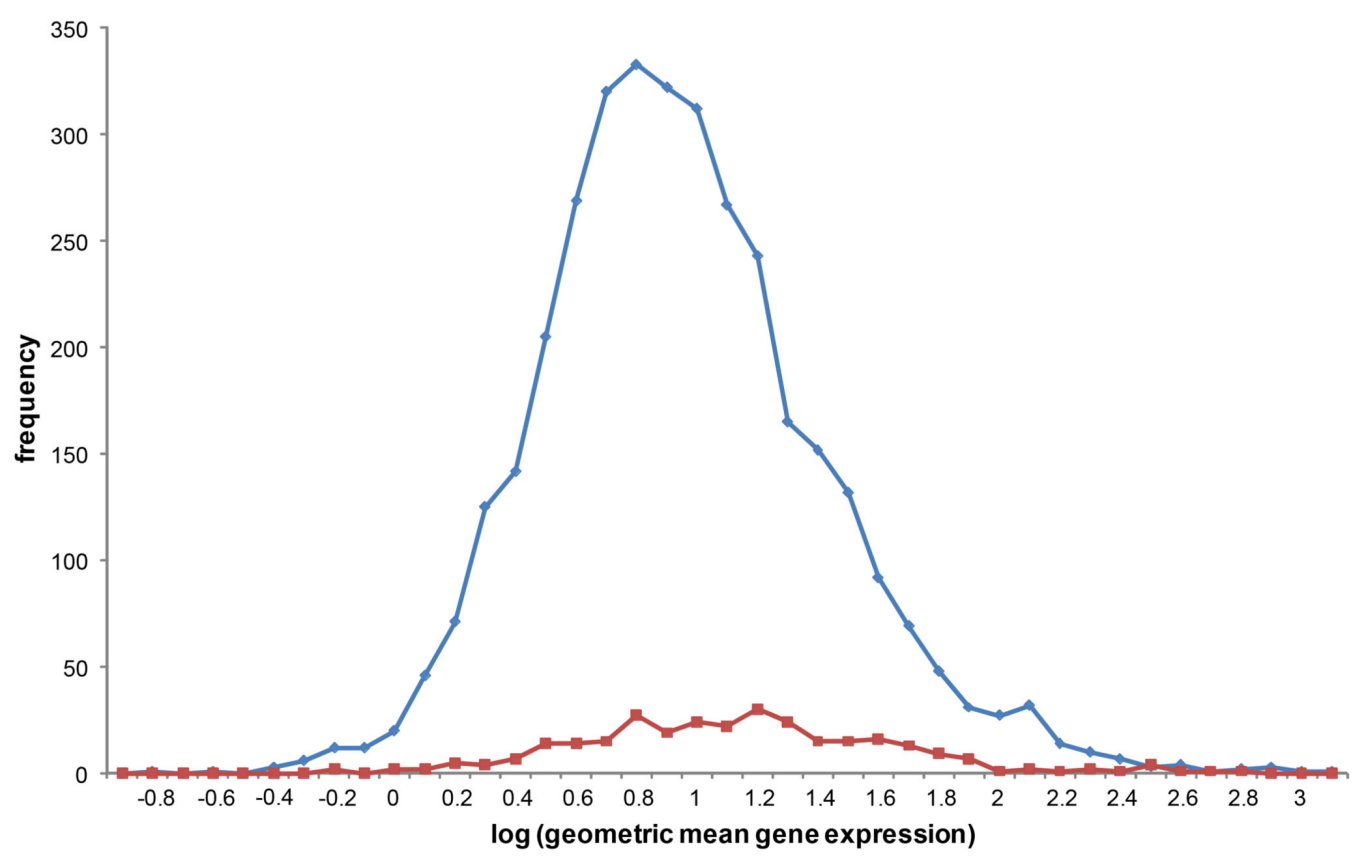
Frequency histogram of relative gene expression for human housekeeping genes Conserved maternal transcripts (COMATs, red line) tend to have a higher gene expression (measured reads per kb per million mapped reads, RPKM) than non-COMATs (blue). However, COMATs still represent several orders of magnitude of gene expression. Gene expression data from Eisenberg & Levanon (2013).

A final concern was that the COMATs are simply conserved genes that tend to be highly expressed, and so are more likely to be detected in non-exhaustive sequencing experiments. We therefore used the expression data of Eisenberg & Levanon (2013) to compare the read depth of these two types of gene (COMATS and non-COMATS) in human tissues. Overall, COMATs tend to be more highly expressed, but they represent a set of genes that have a large range in their quantitative gene expression (Figure 3). Thus, while the mean gene expression in the conserved data set is higher (COMAT mean log geometric gene expression = 1.08, S.E. 0.03; non-COMAT mean = 0.90, S.E. 0.008; *P* < 0.001), the individual variation is considerable in both datasets (S.D. 0.51 and 0.47 respectively). Thus, a lack of depth in sequencing experiments cannot wholly explain the existence of COMATs.

## Discussion

Much excitement has been caused by the discovery that the evolution of gene expression patterns seems to underpin the morphological hourglass pattern of both plants and animals (Kalinka *et al.*, 2010, Meyerowitz, 2002, Quint *et al.*, 2012). Thus, the long-standing observation that vertebrate morphology is at its most conserved during the embryonic pharyngula or phylotypic period is generally mirrored by conserved expression patterns of conserved genes at these stages (Kalinka and Tomancak, 2012, Kalinka *et al.*, 2010). In contrast, active transcription in the early zygote is much more limited. Early animal embryos instead largely rely upon RNAs and proteins provided by the maternal gonad during oocyte maturation. This transcriptionally-quiescent period might, *a priori*, be considered evolutionarily constrained, as the maternally provided transcriptome is widely considered to fulfill one major role, the initiation and management of several rounds of rapid cell division. Every one of these early cell divisions is a critical event that must be faithfully completed to ensure the development of a healthy embryo (Evsikov *et al.*, 2006).

Few studies have investigated the level of conservation of maternally provided genes (Shen-Orr *et al.*, 2010), despite their well-recognised importance in early development (Wieschaus, 1996). Indeed there are few comprehensive datasets of maternally provisioned transcripts even in well-characterised taxa, and none in the Lophotrochozoa. Improvements in sequencing technologies mean that quantitative transcriptome studies are now possible on organisms that lack genomic resources. Our work therefore provides a list of conserved maternal transcripts, or COMATs (Table 6; Supplementary Table 1), that may be useful to the wider community interested in the study of early bilaterian development.

We identified a core set of COMATs from seven representatives of the three bilaterian superphyla, spanning >600 million years of evolution (Peterson *et al.*, 2008). These species display highly divergent modes of development (from direct to indirect, and mosaic to regulative). Since the *L. stagnalis* maternal transcriptome we report here is unlikely to be complete, one possibility is that our estimate of 5-10% of all maternally provisioned transcripts being conserved across the Bilateria may rise upon deeper sampling of the snail transcriptome. Conversely, the number may reduce as maternal transcriptomes from more taxa are included in the analysis.

Unsurprisingly, we found that many of these genes had nucleotide (especially ATP and GTP) binding functions, were associated with protein degradation or had activities associated with the cell cycle (Table 6). The majority of functions ascribed are probably accurately defined as housekeeping (Eisenberg and Levanon, 2013). One possibility is that some of the most conserved maternal RNAs are those that cannot be provided (solely) as proteins. Cell cycle genes may be illustrative, because some cell cycle proteins are degraded every cycle and so maternal protein alone cannot be sufficient. Finally, the fact that the ~32-cell transcriptome was neither enriched nor underrepresented for any gene ontology relative to the 1 to 2-cell transcriptome, along with a relative over-representation of maternal-zygotic transcripts that are conserved between *M. musculus* / *C. elegans* and *L. stagnalis* suggests that the same transcripts are at least still present during early zygotic transcription (Supplementary Figure 1).

Given the wide variety of developmental modes and rates displayed by metazoan embryos, as well as the hourglass theory of evolution (Kalinka and Tomancak, 2012), one view is that we might expect to find relatively few deeply conserved maternal transcripts. Alternatively, as it has been documented that a relatively large fraction (between 45% and 75%) of all genes within a species’ genome can be found as maternal transcripts (see references within Tadros and Lipshitz, 2009), another view is that maternal transcripts that are conserved between different organisms may be a stochastic subset of a large maternal transcriptome. Instead, our analyses suggest that there is a core and specific set of maternal transcripts that may be essential for early cell divisions, irrespective of the precise mode of development.

While both our data and the others utilised in this study have obvious limitations, primarily the limited sequencing coverage, it is thus uncertain whether further investigation will reveal a greater or lesser proportion of conserved maternal transcripts. However, a simultaneous consideration is that we have detected those genes that are conserved and transcribed at a relatively high level across all taxa, since the study is at best partially quantitative. Further studies are warranted to reveal the true nature of this conservation. Nonetheless, as we found that the conserved maternal part of a well annotated group of *H. sapiens* housekeeping genes is enriched for precisely the same functions (Table 6, Supplementary Table 3), we can robustly conclude that there is undoubtedly highly conserved gene expression in the early development of bilaterian embryos. There may also be a distinct set of genes, with mostly housekeeping and nucleotide metabolic functions, that is a necessary starting point of the maternal-to-zygotic transition.

Our analyses thus suggest that the ancestral function of maternal provisioning in animal eggs is to supply the zygote with the materials with which to perform the basic cellular functions of rapid cell division in the early stages of development. The extent of the provisioning is evolutionarily labile, with species that have evolved rapid development relying more on maternal products. Addition of patterning molecules is phylogenetically contingent: as different groups and species have evolved different mechanisms of patterning the embryo and been under selection for fast patterning (as in lineage-driven, or mosaic development) or delayed patterning (as in species with regulative development), so the role of maternal factors in driving patterning has changed.

## Materials and Methods

### cDNA library construction

Early development in the pond snail *L. stagnalis* has been described in exquisite morphological and cytological detail (Raven, 1966). However, the *L. stagnalis* MZT has not been mapped in the same detail as in model species, but transcription from zygotic nuclei was first detected in 8-cell embryos, and major transcriptional activity detected at the 24-cell stage (Morrill, 1982). While division cycles are not as rapid as development in *C. elegans* or *D. melanogaster*, the *L. stagnalis* embryo does not divide for ~3 hour at the 24-cell stage, suggesting this may represent a shift from maternal to zygotic control. We thus separately sampled 1 to 2-cell and ~32-cell stage *L. stagnalis* embryos from a laboratory stock maintained in Nottingham, representing the maternal component and the early stages of zygotic transcription. Zygotes were manually dissected out of their egg capsules and stored in RNAlater (Ambion). As one embryo was expected to yield ~ 0.5 ng RNA, more than one thousand individual embryos of each type were pooled. Total RNA was then extracted using the Qiagen RNeasy Plus Micro Kit. cDNA was then synthesised and two non-normalised cDNA libraries were constructed using the MINT system (Evrogen). The libraries were then processed for sequencing on the Roche 454 FLX platform in the Edinburgh Genomics facility, University of Edinburgh. The raw data have been submitted to the European Nucleotide Archive under bioproject PRJEB7773.

### Transcriptome assembly

The raw Roche 454 data were screened for MINT and sequencing adapters and trimmed of low quality base calls. The reads from each library were assembled using gsAssembler (version 2.6; also known as Newbler; 454 Life Sciences) and MIRA (Chevreux *et al.*, 2004) separately, and then the two assemblies were assembled together using CAP3 (Huang and Madan, 1999), following the proposed best practice for transcriptome assembly from 454 data (Kumar and Blaxter, 2010). gsAssembler assemblies were run with the −cdna and −urt options. MIRA assemblies used job options ‘denovo, est, accurate, 454’ and with clipping by quality off (−CL:qc=no). CD-HIT was then used to remove redundant sequences from the merged CAP3 assemblies (Li and Godzik, 2006), running cd-hit-est with sequence identity threshold 0.98 (−c 0.98) and clustering to most similar cluster (−g 1). The assembly has been made available on afterParty (http://afterparty.bio.ed.ac.uk).

### Maternal transcriptomes from other species

We identified a number of published, high-throughput, maternal transcriptome studies from *Ciona intestinalis* (Urochordata, Deutrostomia), *Danio rerio, Mus musculus*, *Homo sapiens* (Chordata, Deuterostomia), *C. elegans* (Nematoda, Ecdysozoa) and *D. melanogaster* (Arthropoda, Ecdysozoa). A “maternal transcript” is an mRNA that is present in the embryo before the initiation of major zygotic transcription. This does not mean that these mRNAs are not also later also transcribed from the zygotic genome in the developing embryo.

We carried out a reciprocal tBLASTx comparison of the *L. stagnalis* 1 to 2-cell transcriptome against each of the other datasets, using a threshold expect value of 1e^−10^. By identifying *L. stagnalis* transcripts that had homologues in all of the species we identified a putative set of conserved bilaterian maternal transcripts.

### Functional annotation of transcriptome

The 1 to 2-cell and 32-cell transcriptome assemblies were annotated with gene ontology (GO) terms using Blast2GO v 2.7.0 against the NCBI non-redundant (nr) protein database, with an E-value cutoff of 1e-05. GO term distribution was quantified using the Combined Graph function of Blast2GO, with enrichment assessed using the Fisher’s Exact Test function (Conesa *et al.*, 2005).

### In situ validation of representative transcripts

We validated the maternal expression of a selection of sequences in *L. stagnalis* 1-cell embryos by using whole mount *in situ* hybridisation (WMISH). Primers were designed to amplify fragments of selected genes, which were then cloned into pGEM-T and verified by standard Sanger sequencing. Complementary riboprobes were prepared from these templates as described in Jackson *et al.,* (2007a). The WMISH protocol we employed here for *L. stagnalis* is similar to previously described protocols for molluscan embryos and larvae (Jackson *et al.*, 2006, Jackson *et al.*, 2007b) with some important modifications (described elsewhere; in review). The colour reactions for all hybridisations (including the negative β-tubulin control) were allowed to proceed for the same length of time, and all samples cleared in 60% glycerol and imaged under a Zeiss Axio Imager Z1 microscope. The primers used are shown in Table 1.

## Supplementary Material

Supplementary Material

Supplementary Tables

## Figures and Tables

**TABLE 1:** PRIMER SEQUENCES USED TO ISOLATE GENE FRAGMENTS FOR RIBOPROBE SYNTHESES

Gene	Forward primer (5′ to 3′)	Reverse primer (5′ to 3′)
beta-tubulin	TGTGGAATGGATCCCCAACAATGTCA	TCACTCAGGAGCTTTGATACGGCTTG
c2724 ATP-dependent RNA helicase	GCAGCGGTTTCTTCCGCAATG	TTTTTCTCTCCTCTTTACTGCTG
c453 heat shock 70 kda protein	CCACTGCTGCAGCCATTGCCTA	CTGAATGAGCACACCGGGCTGA
c7974 ADP-ribosylation factor 4	CAAGGTGCAACTGCCACGCAAG	AAATCCCACCACCACCCCCAAC
c9053 proteasome alpha 6 subunit	CGCGCTCGCTATGAGGCAGCTA	TCATGGTATCAGCAACACCCACA
c579 ergic and golgi 2	CGTCTGCTACAGGTGGCGGTTTG	TCCGTGGTTGATTGGCCGGTTA
c9016 eukaryotic translation initiation factor 3 subunit i	TGGTGCTGTTTGGTGCATTGATTG	AGCGGGCATCAAATTTGCCAAC
c8075 eukaryotic translation elongation factor	TACTGCGCCAAGCCATTGGTGA	CTGAAGCAGGGCATCACCAGCA
c8318 78 kda glucose-regulated protein	CGCAAAACCAGCGACATATAAGCA	TGGCTGCAGCAGTTGGCTCATT

**TABLE 2:** ASSEMBLY OF THE *LYMNAEA STAGNALIS* EMBRYO TRANSCRIPTOMES

	1 cell transcriptome				32 cell transcriptome			
	Newbler 2.6	MIRA	Merged	Merged + CD-Hit	Newbler 2.6	MIRA	Merged	Merged + CD-Hit
Number of contigs	13,201	15,419	11,222	11,212	11,056	14,422	9,512	9,497
Max contig length	4,258	2,937	6,051	6,051	4,214	3,564	4,212	4,212
Number contigs >100bp	12,908	15,184	11,146	11,136	10,921	14,325	9,490	9,475
>100bp N50	700	630	782	781	847	689	940	938
>100bp GC content	36.3	35.8	36.3	36.3	36.2	35.3	36.2	36.2
Number contigs >1000bp	1,685	1,375	1,869	1,861	2,081	1,843	2,245	2,234
>1000bp N50	1,390	1,317	1,407	1,406	1,520	1,424	1,533	1,533
>1000bp GC content	36.4	36.8	36.4	36.4	36.3	36.5	36.3	36.3
Contigs versus SwissProt hits	27.60%	25.80%	30.90%	30.90%	33.20%	29.20%	36.20%	36.20%

**TABLE 3:** MATERNAL TRANSCRIPTOME DATASETS USED IN THIS STUDY

Taxonomic group / Species	Common name	Number of maternal genes	Method	Source
**Deuterostomia**				
*Homo sapiens*	human	7,470	Array analysis of metaphase II oocytes	Grøndahl et al. 2010
*Mus musculus*	mouse	4,643*	Sanger sequencing of oocyte cDNA library	Evsikov et al. 2006
*Danio rerio*	zebrafish	4,375*	ABI Solid cDNA sequences of oocyte and early embryo	Aanes et al. 2011
*Ciona intestinalis*	*Ciona* / sea squirt	4,041	Array analysis of early embryo	Azumi et al. 2007
**Ecdysozoa**				
*Drosophila melanogaster*	*Drosophila* / fly	6,582#	Array analysis of early embryo	De Renzis et al. 2007
*Caenorhabditis elegans*	*C. elegans* / worm	5,081*	Array analysis of early embryo	Baugh et al. 2003
**Lopphotrochozoa**				
*Lymnaea stagnalis*	snail	11,212	454 sequencing of cDNA library from 1 cell embryo	This study

* more sequences listed in paper, but not all retrievable or present in database (mouse ~5,400; worm 6,042; zebratish 4,465)

# fewer sequences listed in paper compared with database (6,485)

**TABLE 4:** COMPARISON BETWEEN MATERNAL TRANSCRIPTOMES

Species	Maternal transcriptome	Number with orthologues in *Lymnaea stagnalis* transcriptome	%	Unique hits	%	Reciprocal hits	%	Unique reciprocal hits	%
*Homo sapiens*	7,470	2,394	32%	1,852	25%	2,698	36%	1,768	24%
*Mus musculus*	4,643	1,954	42%	1,442	31%	2,013	43%	1,361	29%
*Danio rerio*	4,375	1,913	44%	1,452	33%	1,985	45%	1,328	30%
*Ciona intestinalis*	4,041	1,360	34%	954	24%	1,110	27%	936	23%
*Drosophila melanogaster*	6,582	2,501	38%	1,980	30%	2,903	44%	1,900	29%
*Caenorhabditis elegans*	5,081	1,662	33%	1,220	24%	1,628	32%	1,181	23%

**TABLE 5:** HIGHEST LEVEL GENE ONTOLOGY TERMS ENRICHED IN THE CONSERVED MATERNAL DATASET

GO-ID	Term*	Category	FDR	P-Value after FDR	Number in test group	Number in 1 cell reference	Number in reference total	Number not annotated in test	Number not annotated reference
GO:0005524	ATP binding	F	2.83E-33	5.84E-36	119	136	255	271	1953
GO:0005525	GTP binding	F	2.62E-15	1.08E-17	42	28	70	348	2061
GO:0051082	unfolded protein binding	F	5.10E-11	2.75E-13	24	9	33	366	2080
GO:0008026	ATP-dependent helicase activity	F	6.39E-09	7.10E-11	24	15	39	366	2074
GO:0003924	GTPase activity	F	1.41E-08	1.61E-10	25	18	43	365	2071
GO:0004674	protein serine/threonine kinase activity	F	4.92E-08	6.17E-10	25	20	45	365	2069
GO:0003755	peptidyl-prolyl cis-trans isomerase activity	F	5.29E-07	7.72E-09	14	4	18	376	2085
GO:0004767	sphingomyelin phosphodiesterase activity	F	1.05E-04	2.28E-06	7	0	7	383	2089
GO:0004298	threonine-type endopeptidase activity	F	1.21E-04	2.74E-06	8	1	9	382	2088
GO:0004842	ubiquitin-protein ligase activity	F	1.09E-03	2.96E-05	15	17	32	375	2072
GO:0005200	structural constituent of cytoskeleton	F	2.95E-03	8.91E-05	6	1	7	384	2088
GO:0008568	microtubule-severing ATPase activity	F	3.06E-03	9.43E-05	5	0	5	385	2089
GO:0042288	MHC class I protein binding	F	1.50E-02	6.05E-04	4	0	4	386	2089
GO:0005528	FK506 binding	F	1.50E-02	6.05E-04	4	0	4	386	2089
GO:0019899	enzyme binding	F	1.92E-02	8.08E-04	24	56	80	366	2033
GO:0003676	nucleic acid binding	F	2.13E-02	9.21E-04	80	293	373	310	1796
GO:0007264	small GTPase mediated signal transduction	P	1.78E-10	1.24E-12	25	12	37	365	2077
GO:0051258	protein polymerization	P	2.72E-07	3.75E-09	19	11	30	371	2078
GO:0006184	GTP catabolic process	P	8.66E-07	1.32E-08	24	23	47	366	2066
GO:0000413	protein peptidyl-prolyl isomerization	P	2.30E-06	3.94E-08	13	4	17	377	2085
GO:0006468	protein phosphorylation	P	2.76E-06	4.87E-08	34	52	86	356	2037
GO:0006200	ATP catabolic process	P	5.78E-04	1.50E-05	16	18	34	374	2071
GO:0031145	anaphase-promoting complex-dependent proteasomal ubiquitin-dependent protein catabolic process	P	1.83E-03	5.19E-05	9	5	14	381	2084
GO:0000209	protein polyubiquitination	P	2.90E-03	8.70E-05	12	12	24	378	2077
GO:0031110	regulation of microtubule polymerization or depolymerization	P	3.06E-03	9.43E-05	5	0	5	385	2089
GO:0000165	MAPK cascade	P	3.12E-03	9.69E-05	8	4	12	382	2085
GO:0030174	regulation of DNA-dependent DNA replication initiation	P	3.12E-03	9.69E-05	8	4	12	382	2085
GO:0045087	innate immune response	P	3.49E-03	1.11E-04	10	8	18	380	2081
GO:0051437	positive regulation of ubiquitin-protein ligase activity involved in mitotic cell cycle	P	5.31E-03	1.77E-04	7	3	10	383	2086
GO:0007018	microtubule-based movement	P	6.73E-03	2.30E-04	12	14	26	378	2075
GO:0031346	positive regulation of cell projection organization	P	8.65E-03	3.09E-04	6	2	8	384	2087
GO:0051495	positive regulation of cytoskeleton organization	P	8.65E-03	3.09E-04	6	2	8	384	2087
GO:0000216	M/G1 transition of mitotic cell cycle	P	1.13E-02	4.21E-04	7	4	11	383	2085
GO:0051084	’*de novo*’ post-translational protein folding	P	1.29E-02	4.92E-04	5	1	6	385	2088
GO:0000084	S phase of mitotic cell cycle	P	1.45E-02	5.71E-04	10	11	21	380	2078
GO:0008356	asymmetric cell division	P	1.50E-02	6.05E-04	4	0	4	386	2089
GO:0010458	exit from mitosis	P	1.50E-02	6.05E-04	4	0	4	386	2089
GO:0071363	cellular response to growth factor stimulus	P	1.69E-02	6.97E-04	9	9	18	381	2080
GO:0051704	multi-organism process	P	2.41E-02	1.05E-03	25	61	86	365	2028
GO:0051225	spindle assembly	P	3.17E-02	1.50E-03	5	2	7	385	2087
GO:0050684	regulation of mRNA processing	P	3.17E-02	1.50E-03	5	2	7	385	2087
GO:0006977	DNA damage response, signal transduction by p53 class mediator resulting in cell cycle arrest	P	3.17E-02	1.50E-03	5	2	7	385	2087
GO:0007167	enzyme linked receptor protein signaling pathway	P	3.31E-02	1.58E-03	12	19	31	378	2070
GO:0051436	negative regulation of ubiquitin-protein ligase activity involved in mitotic cell cycle	P	3.61E-02	1.75E-03	6	4	10	384	2085
GO:0030522	intracellular receptor signaling pathway	P	4.64E-02	2.29E-03	8	9	17	382	2080
GO:0045664	regulation of neuron differentiation	P	4.64E-02	2.29E-03	8	9	17	382	2080
GO:0005874	microtubule	C	3.31E-06	5.93E-08	21	19	40	369	2070
GO:0019773	proteasome core complex, alpha-subunit complex	C	1.05E-04	2.28E-06	7	0	7	383	2089
GO:0045298	tubulin complex	C	3.06E-03	9.43E-05	5	0	5	385	2089
GO:0005681	spliceosomal complex	C	5.33E-03	1.78E-04	18	30	48	372	2059
GO:0048471	perinuclear region of cytoplasm	C	1.69E-02	7.00E-04	11	14	25	379	2075
GO:0005829	cytosol	C	2.00E-02	8.53E-04	42	126	168	348	1963
GO:0005663	DNA replication factor C complex	C	3.17E-02	1.50E-03	5	2	7	385	2087

* ordered by category and significance

**TABLE 6:** THE 300 HUMAN GENES IN THE CONSERVED MATERNAL DATASET

Gene	Accession	Description	Gene	Accession	Description
MTRR	NM_002454	5-methyltetrahydrofolate-homocysteine methyltransferase reductase	NOP5/NOP58	NM_015934	Nucleolar protein NOP5/NOP58
ACAD9	NM_014049	Acyl-Coenzyme A dehydrogenase family, member 9	NAP1L4	NM_005969	Nucleosome assembly protein 1-like 4
ACADVL	NM_000018	Acyl-Coenzyme A dehydrogenase, very long chain	OTUB1	NM_017670	OTU domain, ubiquitin aldehyde binding 1
ARF1	NM_001658	ADP-ribosylation factor 1	OSBPL2	NM_014835	Oxysterol binding protein-like 2
ARF5	NM_001662	ADP-ribosylation factor 5	PAK2	NM_002577	P21 (CDKN1A)-activated kinase 2
ARF6	NM_001663	ADP-ribosylation factor 6	PCAF	NM_003884	P300/CBP-associated factor
ARFGAP3	NM_014570	ADP-ribosylation factor GTPase activating protein 3	PCTK1	NM_006201	PCTAIRE protein kinase 1
ARL1	NM_001177	ADP-ribosylation factor-like 1	PPWD1	NM_015342	Peptidylprolyl isomerase domain and WD repeat containing 1
AHSA1	NM_012111	AHA1, activator of heat shock 90kDa protein ATPase homolog 1 (yeast)	PPIE	NM_006112	Peptidylprolyl isomerase E (cyclophilin E)
ALDH9A1	NM_000696	Aldehyde dehydrogenase 9 family, member A1	PPIF	NM_005729	Peptidylprolyl isomerase F (cyclophilin F)
AAMP	NM_001087	Angio-associated, migratory cell protein	PPIH	NM_006347	Peptidylprolyl isomerase H (cyclophilin H)
ANKRD17	NM_032217	Ankyrin repeat domain 17	PRDX1	NM_002574	Peroxiredoxin 1
ANKRD28	NM_001195098	Ankyrin repeat domain 28	PRDX2	NM_005809	Peroxiredoxin 2
ARD1A	NM_003491	ARD1 homolog A, N-acetyltransferase (S. cerevisiae)	PECI	NM_006117	Peroxisomal D3,D2-enoyl-CoA isomerase
ACTR1A	NM_005736	ARP1 actin-related protein 1 homolog A, centractin alpha (yeast)	PI4KB	NM_002651	Phosphatidylinositol 4-kinase, catalytic, beta
ACTR1B	NM_005735	ARP1 actin-related protein 1 homolog B, centractin beta (yeast)	PLAA	NM_001031689	Phospholipase A2-activating protein
ARNT	NM_001668	Aryl hydrocarbon receptor nuclear translocator	PRPSAP1	NM_002766	Phosphoribosyl pyrophosphate synthetase-associated protein 1
ATP5A1	NM_004046	ATP synthase, H+ transporting, mitochondrial F1 complex, alpha subunit 1	PAFAH1B1	NM_000430	Platelet-activating factor acetylhydrolase, isoform Ib, alpha subunit 45kDa
ATP5B	NM_001686	ATP synthase, H+ transporting, mitochondrial F1 complex, beta polypeptide	PLRG1	NM_002669	Pleiotropic regulator 1 (PRL1 homolog, Arabidopsis)
ATAD1	NM_032810	ATPase family, AAA domain containing 1	PHB	NM_002634	Prohibitin
ABCB10	NM_012089	ATP-binding cassette, sub-family B (MDR/TAP), member 10	PHB2	NM_001144831	Prohibitin 2
ABCB7	NM_004299	ATP-binding cassette, sub-family B (MDR/TAP), member 7	PSMC2	NM_002803	Proteasome (prosome, macropain) 26S subunit, ATPase, 2
BXDC5	NM_025065	Brix domain containing 5	PSMC3	NM_002804	Proteasome (prosome, macropain) 26S subunit, ATPase, 3
BRD7	NM_013263	Bromodomain containing 7	PSMC4	NM_006503	Proteasome (prosome, macropain) 26S subunit, ATPase, 4
BPTF	NM_004459	Bromodomain PHD finger transcription factor	PSMC5	NM_002805	Proteasome (prosome, macropain) 26S subunit, ATPase, 5
BUB3	NM_004725	BUB3 budding uninhibited by benzimidazoles 3 homolog (yeast)	PSMC6	NM_002806	Proteasome (prosome, macropain) 26S subunit, ATPase, 6
CAB39	NM_016289	Calcium binding protein 39	PSMD10	NM_002814	Proteasome (prosome, macropain) 26S subunit, non-ATPase, 10
CALU	NM_001219	Calumenin	PSMD11	NM_002815	Proteasome (prosome, macropain) 26S subunit, non-ATPase, 11
CBR4	NM_032783	Carbonyl reductase 4	PSMA1	NM_002786	Proteasome (prosome, macropain) subunit, alpha type, 1
CSNK1A1	NM_001892	Casein kinase 1, alpha 1	PSMA2	NM_002787	Proteasome (prosome, macropain) subunit, alpha type, 2
CSNK1D	NM_001893	Casein kinase 1, delta	PSMA3	NM_002788	Proteasome (prosome, macropain) subunit, alpha type, 3
CSNK2A3	NM_001256686	casein kinase 2, alpha 3 polypeptide	PSMA4	NM_002789	Proteasome (prosome, macropain) subunit, alpha type, 4
CTCF	NM_006565	CCCTC-binding factor (zinc finger protein)	PSMA5	NM_002790	Proteasome (prosome, macropain) subunit, alpha type, 5
CNBP	NM_003418	CCHC-type zinc finger, nucleic acid binding protein	PSMA6	NM_002791	Proteasome (prosome, macropain) subunit, alpha type, 6
CD63	NM_001780	CD63 molecule	PSMA7	NM_002792	Proteasome (prosome, macropain) subunit, alpha type, 7
CRKRS	NM_015083	CDC2-related kinase, arginine/serine-rich	PSMB2	NM_002794	Proteasome (prosome, macropain) subunit, beta type, 2
CDC37	NM_007065	CDC37 homolog (S. cerevisiae)	PSMB6	NM_002798	Proteasome (prosome, macropain) subunit, beta type, 6
CDC42	NM_001791	CDC42 (GTP binding protein, 25kDa)	PSMB7	NM_002799	Proteasome (prosome, macropain) subunit, beta type, 7
CDC5L	NM_001253	CDC5 CDC5-like (S. pombe)	PIAS1	NM_016166	Protein inhibitor of activated STAT, 1
CLK3	NM_003992	CDC-like kinase 3	PRKAA1	NM_006251	Protein kinase, AMP-activated, alpha 1 catalytic subunit
CCT3	NM_005998	Chaperonin containing TCP1, subunit 3 (gamma)	PPP1CC	NM_002710	Protein phosphatase 1, catalytic subunit, gamma isoform
CCT4	NM_006430	Chaperonin containing TCP1, subunit 4 (delta)	PPP2CB	NM_001009552	Protein phosphatase 2 (formerly 2A), catalytic subunit, beta isoform
CCT5	NM_012073	Chaperonin containing TCP1, subunit 5 (epsilon)	PPP2R5D	NM_006245	Protein phosphatase 2, regulatory subunit B’, delta isoform
CCT6A	NM_001762	Chaperonin containing TCP1, subunit 6A (zeta 1)	PPP4C	NM_002720	Protein phosphatase 4 (formerly X), catalytic subunit
CCT7	NM_006429	Chaperonin containing TCP1, subunit 7 (eta)	PPP6C	NM_002721	Protein phosphatase 6, catalytic subunit
CCT8	NM_006585	Chaperonin containing TCP1, subunit 8 (theta)	PSKH1	NM_006742	Protein serine kinase H1
CHD4	NM_001273	Chromodomain helicase DNA binding protein 4	PTPN1	NM_002827	Protein tyrosine phosphatase, non-receptor type 1
C14orf130	NM_175748	Chromosome 14 open reading frame 130	PRPF31	NM_015629	PRP31 pre-mRNA processing factor 31 homolog (S. cerevisiae)
CSTF1	NM_001324	Cleavage stimulation factor, 3′ pre-RNA, subunit 1, 50kDa	PRPF4	NM_004697	PRP4 pre-mRNA processing factor 4 homolog (yeast)
CSTF2T	NM_015235	Cleavage stimulation factor, 3′ pre-RNA, subunit 2, 64kDa, tau variant	PWP2	NM_005049	PWP2 periodic tryptophan protein homolog (yeast)
COPA	NM_004371	Coatomer protein complex, subunit alpha	RAB10	NM_016131	RAB10, member RAS oncogene family
COPS2	NM_004236	COP9 constitutive photomorphogenic homolog subunit 2 (Arabidopsis)	RAB11B	NM_004218	RAB11B, member RAS oncogene family
CTDSP2	NM_005730	CTD (carboxy-terminal domain, RNA polymerase II, polypeptide A) small phosphatase 2	RAB14	NM_016322	RAB14, member RAS oncogene family
CLEC3B	NM_015004	C-type lectin domain family 3, member B	RAB18	NM_021252	RAB18, member RAS oncogene family
CUL1	NM_003592	Cullin 1	RAB1A	NM_004161	RAB1A, member RAS oncogene family
CUL4B	NM_003588	Cullin 4B	RAB2A	NM_002865	RAB2A, member RAS oncogene family
CDK9	NM_001261	Cyclin-dependent kinase 9	RAB5C	NM_004583	RAB5C, member RAS oncogene family
CYB5B	NM_030579	Cytochrome b5 type B (outer mitochondrial membrane)	RAB7A	NM_004637	RAB7A, member RAS oncogene family
CYP2U1	NM_183075	Cytochrome P450, family 2, subfamily U, polypeptide 1	RDX	NM_002906	Radixin
DAZAP1	NM_018959	DAZ associated protein 1	RANBP1	NM_002882	RAN binding protein 1
DDX19B	NM_007242	DEAD (Asp-Glu-Ala-As) box polypeptide 19B	RAN	NM_006325	RAN, member RAS oncogene family
DDX1	NM_004939	DEAD (Asp-Glu-Ala-Asp) box polypeptide 1	RAP1A	NM_002884	RAP1A, member of RAS oncogene family
DDX17	NM_006386	DEAD (Asp-Glu-Ala-Asp) box polypeptide 17	RHOA	NM_001664	Ras homolog gene family, member A
DDX18	NM_006773	DEAD (Asp-Glu-Ala-Asp) box polypeptide 18	REST	NM_005612	RE1-silencing transcription factor
DDX21	NM_004728	DEAD (Asp-Glu-Ala-Asp) box polypeptide 21	RFC2	NM_002914	Replication factor C (activator 1) 2, 40kDa
DDX23	NM_004818	DEAD (Asp-Glu-Ala-Asp) box polypeptide 23	RFC5	NM_007370	Replication factor C (activator 1) 5, 36.5kDa
DDX24	NM_020414	DEAD (Asp-Glu-Ala-Asp) box polypeptide 24	RBBP4	NM_005610	Retinoblastoma binding protein 4
DDX27	NM_017895	DEAD (Asp-Glu-Ala-Asp) box polypeptide 27	RXRA	NM_002957	Retinoid X receptor, alpha
DDX3X	NM_001356	DEAD (Asp-Glu-Ala-Asp) box polypeptide 3, X-linked	RDH14	NM_020905	Retinol dehydrogenase 14 (all-trans/9-cis/11-cis)
DDX41	NM_016222	DEAD (Asp-Glu-Ala-Asp) box polypeptide 41	REXO1	NM_020695	REX1, RNA exonuclease 1 homolog (S. cerevisiae)
DDX47	NM_016355	DEAD (Asp-Glu-Ala-Asp) box polypeptide 47	RPL14	NM_003973	Ribosomal protein L14
DDX54	NM_024072	DEAD (Asp-Glu-Ala-Asp) box polypeptide 54	RPL35	NM_007209	Ribosomal protein L35
DDX56	NM_019082	DEAD (Asp-Glu-Ala-Asp) box polypeptide 56	RPS6KB1	NM_003161	Ribosomal protein S6 kinase, 70kDa, polypeptide 1
DHX15	NM_001358	DEAH (Asp-Glu-Ala-His) box polypeptide 15	RPS6KB2	NM_003952	Ribosomal protein S6 kinase, 70kDa, polypeptide 2
DHX38	NM_014003	DEAH (Asp-Glu-Ala-His) box polypeptide 38	RPS6KA3	NM_004586	Ribosomal protein S6 kinase, 90kDa, polypeptide 3
DHX8	NM_004941	DEAH (Asp-Glu-Ala-His) box polypeptide 8	RRP1	NM_003683	Ribosomal RNA processing 1 homolog (S. cerevisiae)
DHRS7B	NM_015510	Dehydrogenase/reductase (SDR family) member 7B	AHCY	NM_000687	S-adenosylhomocysteine hydrolase
DLG1	NM_004087	Discs, large homolog 1 (*Drosophila*)	SCRIB	NM_015356	Scribbled homolog (*Drosophila*)
DNAJA2	NM_005880	DNAJ (Hsp40) homolog, subfamily A, member 2	STRAP	NM_007178	Serine/threonine kinase receptor associated protein
DNAJA3	NM_005147	DNAJ (Hsp40) homolog, subfamily A, member 3	SETD8	NM_020382	SET domain containing (lysine methyltransferase) 8
DNAJB12	NM_017626	DNAJ (Hsp40) homolog, subfamily B, member 12	SMAD5	NM_005903	SMAD family member 5
DNAJC10	NM_018981	DNAJ (Hsp40) homolog, subfamily C, member 10	SMU1	NM_018225	Smu-1 suppressor of mec-8 and unc-52 homolog (C. elegans)
DNAJC17	NM_018163	DNAJ (Hsp40) homolog, subfamily C, member 17	SHOC2	NM_007373	Soc-2 suppressor of clear homolog (C. elegans)
DNAJC5	NM_025219	DNAJ (Hsp40) homolog, subfamily C, member 5	SLC25A11	NM_003562	Solute carrier family 25 (mitochondrial carrier; oxoglutarate carrier), member 11
DUSP16	NM_030640	Dual specificity phosphatase 16	SLC25A39	NM_016016	Solute carrier family 25, member 39
ELAVL1	NM_001419	ELAV (embryonic lethal, abnormal vision, *Drosophila*)-like 1 (Hu antigen R)	SLC39A7	NM_006979	Solute carrier family 39 (zinc transporter), member 7
ETFA	NM_000126	Electron-transfer-flavoprotein, alpha polypeptide (glutaric aciduria II)	SPG7	NM_003119	Spastic paraplegia 7 (pure and complicated autosomal recessive)
ECHS1	NM_004092	Enoyl Coenzyme A hydratase, short chain, 1, mitochondrial	SPATA5L1	NM_024063	Spermatogenesis associated 5-like 1
ERGIC2	NM_016570	ERGIC and golgi 2	SFRS2	NM_003016	Splicing factor, arginine/serine-rich 2
EEF2	NM_001961	Eukaryotic translation elongation factor 2	SAE1	NM_005500	SUMO1 activating enzyme subunit 1
EIF2AK3	NM_004836	Eukaryotic translation initiation factor 2-alpha kinase 3	UBA2	NM_005499	SUMO1 activating enzyme subunit 2
EIF3D	NM_003753	Eukaryotic translation initiation factor 3, subunit D	TAF5L	NM_014409	TAF5-like RNA polymerase II, p300/CBP-associated factor (PCAF)-associated factor, 65kDa
EIF3I	NM_003757	Eukaryotic translation initiation factor 3, subunit I	TNKS2	NM_025235	Tankyrase, TRF1-interacting ankyrin-related ADP-ribose polymerase 2
EIF4A1	NM_001416	Eukaryotic translation initiation factor 4A, isoform 1	TCP1	NM_030752	T-complex 1
EIF4A3	NM_014740	Eukaryotic translation initiation factor 4A, isoform 3	TXN2	NM_012473	Thioredoxin 2
EIF4E2	NM_004846	Eukaryotic translation initiation factor 4E family member 2	TXNDC9	NM_005783	Thioredoxin domain containing 9
FBXW11	NM_012300	F-box and WD repeat domain containing 11	TIAL1	NM_003252	TIA1 cytotoxic granule-associated RNA binding protein-like 1
FZR1	NM_016263	Fizzy/CDC20 related 1 (*Drosophila*)	TRAP1	NM_001272049	TNF receptor-associated protein 1
FKBP3	NM_002013	FK506 binding protein 3, 25kDa	TOMM70A	NM_014820	Translocase of outer mitochondrial membrane 70 homolog A (S. cerevisiae)
					
FTSJ1	NM_012280	FtsJ homolog 1 (E. coli)	TPI1	NM_000365	Triosephosphate isomerase 1
FUSIP1	NM_006625	FUS interacting protein (serine/arginine-rich) 1	TUFM	NM_003321	Tu translation elongation factor, mitochondrial
GTF2B	NM_001514	General transcription factor IIB	TUBA1B	NM_006082	Tubulin, alpha 1b
GNPDA1	NM_005471	Glucosamine-6-phosphate deaminase 1	TUBA1C	NM_032704	Tubulin, alpha 1c
GRWD1	NM_031485	Glutamate-rich WD repeat containing 1	TUBB	NM_178014	Tubulin, beta
GRPEL1	NM_025196	GrpE-like 1, mitochondrial (E. coli)	YWHAB	NM_003404	Tyrosine 3-monooxygenase/tryptophan 5-monooxygenase activation protein, beta polypeptide
					
GTPBP4	NM_012341	GTP binding protein 4	YWHAE	NM_006761	Tyrosine 3-monooxygenase/tryptophan 5-monooxygenase activation protein, epsilon polypeptide
					
GTPBP10	NM_033107	GTP-binding protein 10 (putative)	UBA52	NM_003333	Ubiquitin A-52 residue ribosomal protein fusion product 1
GNL2	NM_013285	Guanine nucleotide binding protein-like 2 (nucleolar)	UBB	NM_018955	Ubiquitin B
GNL3	NM_014366	Guanine nucleotide binding protein-like 3 (nucleolar)	UBC	NM_021009	Ubiquitin C
H2AFV	NM_012412	H2A histone family, member V	UBE3C	NM_014671	Ubiquitin protein ligase E3C
HBS1L	NM_006620	HBS1-like (S. cerevisiae)	UBA3	NM_003968	Ubiquitin-activating enzyme E1C (UBA3 homolog, yeast)
HSPE1	NM_001202485	Heat shock 10kDa protein 1 (chaperonin 10)	UBE2V1	NM_021988	Ubiquitin-conjugating enzyme E2 variant 1
HSPA5	NM_005347	Heat shock 70kDa protein 5 (glucose-regulated protein, 78kDa)	UBE2A	NM_003336	Ubiquitin-conjugating enzyme E2A (RAD6 homolog)
HSPA8	NM_006597	Heat shock 70kDa protein 8	UBE2B	NM_003337	Ubiquitin-conjugating enzyme E2B (RAD6 homolog)
HSPA9	NM_004134	Heat shock 70kDa protein 9 (mortalin)	UBE2D2	NM_003339	Ubiquitin-conjugating enzyme E2D 2 (UBC4/5 homolog, yeast)
HGS	NM_004712	Hepatocyte growth factor-regulated tyrosine kinase substrate	UBE2D3	NM_003340	Ubiquitin-conjugating enzyme E2D 3 (UBC4/5 homolog, yeast)
HNRPD	NM_002138	Heterogeneous nuclear ribonucleoprotein D (AU-rich element RNA binding protein 1)	UBE2G2	NM_003343	Ubiquitin-conjugating enzyme E2G 2 (UBC7 homolog, yeast)
					
HAT1	NM_003642	Histone acetyltransferase 1	UBE2I	NM_003345	Ubiquitin-conjugating enzyme E2I (UBC9 homolog, yeast)
BAT1	NM_004640	HLA-B associated transcript 1	UBE2N	NM_003348	Ubiquitin-conjugating enzyme E2N (UBC13 homolog, yeast)
IMP4	NM_033416	IMP4, U3 small nucleolar ribonucleoprotein, homolog (yeast)	UBE2Q1	NM_017582	Ubiquitin-conjugating enzyme E2Q (putative) 1
JAK1	NM_002227	Janus kinase 1 (a protein tyrosine kinase)	UBE2R2	NM_017811	Ubiquitin-conjugating enzyme E2R 2
KPNA1	NM_002264	Karyopherin alpha 1 (importin alpha 5)	VRK2	NM_006296	Vaccinia related kinase 2
KLHL8	NM_020803	Kelch-like 8 (*Drosophila*)	VPS4A	NM_013245	Vacuolar protein sorting 4 homolog A (S. cerevisiae)
L3MBTL2	NM_031488	L(3)mbt-like 2 (*Drosophila*)	AKT1	NM_005163	V-akt murine thymoma viral oncogene homolog 1
LRRC47	NM_020710	Leucine rich repeat containing 47	VCP	NM_007126	Valosin-containing protein
MAPRE2	NM_014268	Microtubule-associated protein, RP/EB family, member 2	VBP1	NM_003372	Von Hippel-Lindau binding protein 1
MCM7	NM_005916	Minichromosome maintenance complex component 7	RALA	NM_005402	V-ral simian leukemia viral oncogene homolog A (ras related)
MRPL4	NM_015956	Mitochondrial ribosomal protein L4	WDR12	NM_018256	WD repeat domain 12
MAPK1	NM_002745	Mitogen-activated protein kinase 1	WDR3	NM_006784	WD repeat domain 3
MAPK9	NM_002752	Mitogen-activated protein kinase 9	WDR57	NM_004814	WD repeat domain 57 (U5 snRNP specific)
MAP2K1	NM_002755	Mitogen-activated protein kinase kinase 1	WDR5B	NM_019069	WD repeat domain 5B
MAP2K2	NM_030662	Mitogen-activated protein kinase kinase 2	WDR61	NM_025234	WD repeat domain 61
MAP2K5	NM_002757	Mitogen-activated protein kinase kinase 5	YPEL2	NM_001005404	Yippee-like 2 (*Drosophila*)
MAP4K4	NM_004834	Mitogen-activated protein kinase kinase kinase kinase 4	YME1L1	NM_014263	YME1-like 1 (S. cerevisiae)
MAPKAPK2	NM_004759	Mitogen-activated protein kinase-activated protein kinase 2	YY1	NM_003403	YY1 transcription factor
MLH1	NM_000249	MutL homolog 1, colon cancer, nonpolyposis type 2 (E. coli)	ZBTB6	NM_006626	Zinc finger and BTB domain containing 6
MLLT1	NM_005934	Myeloid/lymphoid or mixed-lineage leukemia (trithorax homolog, *Drosophila*); translocated to, 1	ZNF138	NM_001271649	zinc finger protein 138
MYNN	NM_018657	Myoneurin	ZNF195	NM_007152	Zinc finger protein 195
MYO1E	NM_004998	Myosin IE	ZNF197	NM_006991	Zinc finger protein 197
MTMR1	NM_003828	Myotubularin related protein 1	ZNF289	NM_032389	Zinc finger protein 289, ID1 regulated
NDUFS8	NM_002496	NADH dehydrogenase (ubiquinone) Fe-S protein 8, 23kDa (NADH-coenzyme Q reductase)	ZNF347	NM_032584	Zinc finger protein 347
NEDD8	NM_006156	Neural precursor cell expressed, developmentally down- regulated 8	ZNF37A	NM_003421	Zinc finger protein 37A
NF2	NM_000268	Neurofibromin 2 (bilateral acoustic neuroma)	ZNF397	NM_001135178	Zinc finger protein 397
NHP2L1	NM_005008	NHP2 non-histone chromosome protein 2-like 1 (S. cerevisiae)	ZNF41	NM_007130	Zinc finger protein 41
NEK4	NM_003157	NIMA (never in mitosis gene a)-related kinase 4	ZNF506	NM_001099269	Zinc finger protein 506
NSUN2	NM_017755	NOL1/NOP2/Sun domain family, member 2	ZNF91	NM_003430	Zinc finger protein 91
NOL1	NM_006170	Nucleolar protein 1, 120kDa	ZFAND1	NM_024699	Zinc finger, AN1-type domain 1
NOL5A	NM_006392	Nucleolar protein 5A (56kDa with KKE/D repeat)	ZFAND5	NM_006007	Zinc finger, AN1-type domain 5
NOLA2	NM_017838	Nucleolar protein family A, member 2 (H/ACA small nucleolar RNPs)	ZDHHC5	NM_015457	Zinc finger, DHHC-type containing 5
NOLA3	NM_018648	Nucleolar protein family A, member 3 (H/ACA small nucleolar RNPs)	ZRF1	NM_014377	Zuotin related factor 1

## Abbreviations used in this paper

bp: base pair
COMAT: conserved maternal transcript
GO: gene ontology
MBT: midblastula transition
MZT: maternal-zygotic transition
