# Supplementary material for: A conserved set of maternal genes? Insights from a molluscan transcriptome
Source: Int J Dev Biol. Author manuscript; Available in PMC 2015 Oct 6. (PMC4594767; doi:10.1387/ijdb.140121ad)
Supplement: Supplementary Material [file NIHMS65388-supplement-Supplementary_Material.pdf]

THE INTERNATIONAL JOURNAL OF DEVELOPMENTAL BIOLOGY
www.intjdevbiol.com

doi: 10.1387/ijdb.140121ad

SUPPLEMENTARY MATERIAL

corresponding to:

A conserved set of maternal genes? Insights from a molluscan transcriptome

M. MAUREEN LIU, JOHN W. DAVEY, DANIEL J. JACKSON, MARK L. BLAXTER and ANGUS DAVISON

***Address correspondence to:** Angus Davison. School of Life Sciences, University of Nottingham, University Park, Nottingham, UK.
E-mail: angus.davison@nottingham.ac.uk

Full text for this paper is available at: http://dx.doi.org/10.1387/ijdb.140121ad

Final, author-corrected PDF published online: 28 November 2014.

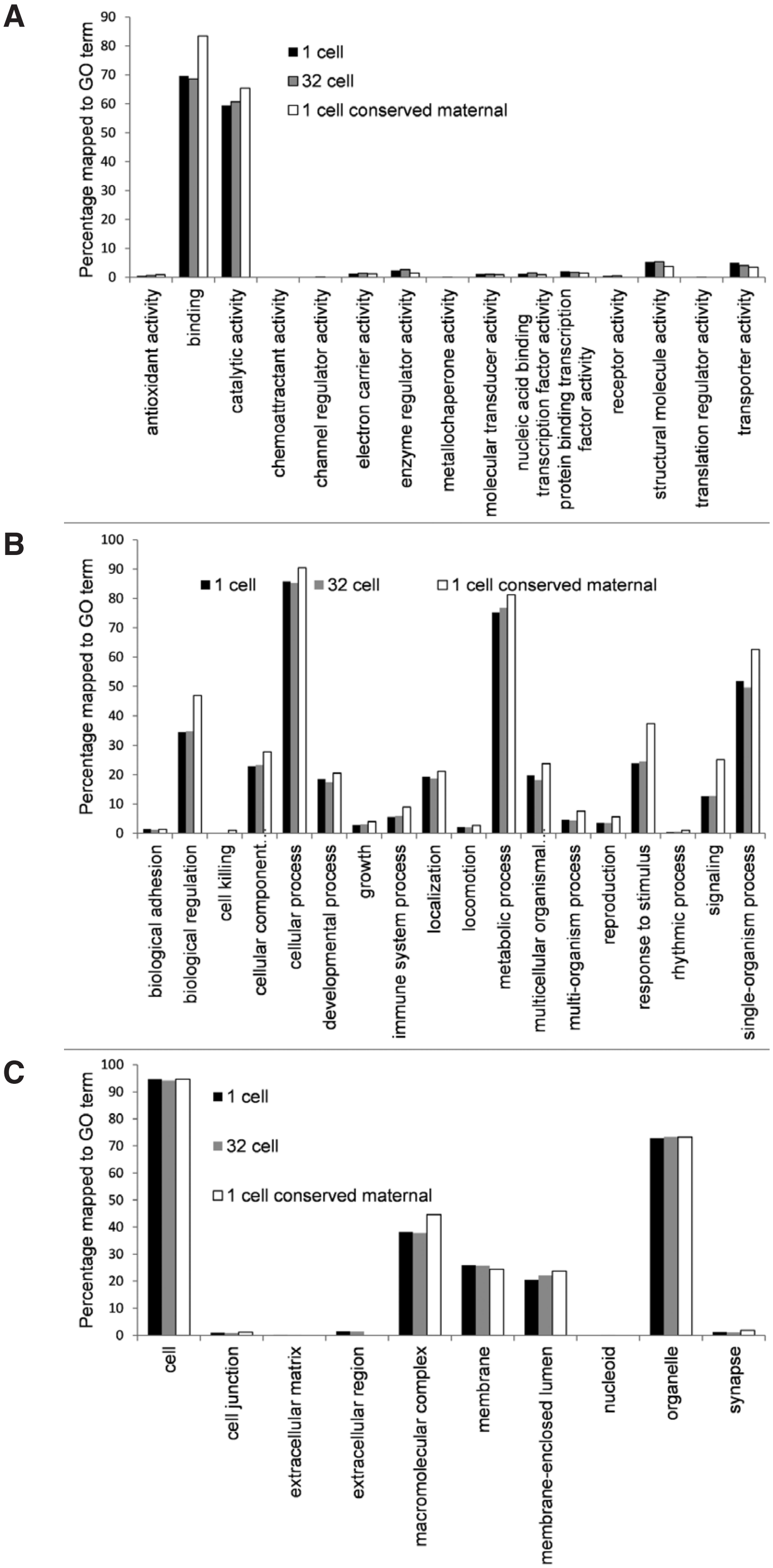

Supplementary Figure 1. Gene Ontology (GO) annotation of *Lymnaea stagnalis* embryonic transcriptomes. *The distribution of GO annotations into functional categories shows no qualitative differences between the 1 to 2-cell and ~32-cell transcriptomes. The 1 to 2-cell conserved bilaterian maternal transcripts (COMAT) are qualitatively enriched for functions relating to binding and catalytic activity* **(A)**, *several biological processes* **(B)**, *as well as macromolecular complex* **(C)**.

Supplementary Table 1. The 481 conserved maternal transcript sequences.

Supplementary Table 2. Gene ontology (GO) terms that are enriched in the conserved maternal transcript dataset.

Supplementary Table 3. GO terms that are enriched in the conserved *Homo sapiens* maternal transcript housekeeping dataset.

Supplementary Table 4. Highest level GO terms enriched in the conserved human housekeeping dataset.

Supplementary Table 1: The 481 conserved maternal transcript sequences

Sequence ID	Sequence description
Contig10233	14-3-3 protein
Contig5003	14-3-3 protein beta alpha
Contig1640	14-3-3 protein epsilon-like
Contig562	14-3-3 zeta
Contig704	26s protease regulatory subunit 4
Contig10345	26s protease regulatory subunit 6a
Contig8089	26s protease regulatory subunit 6a
Contig8981	26s protease regulatory subunit 6b
Contig9889	26s protease regulatory subunit 7-like
Contig612	26s protease regulatory subunit 8-like
Contig200	26s protease regulatory subunit s10b
Contig9140	39s ribosomal protein mitochondrial isoform a
Contig10114	3-phosphoinositide-dependent protein kinase 1-like
Contig4859	3-phosphoinositide-dependent protein kinase 1-like
Contig1526	40s ribosomal protein
Contig173	46 kda fk506-binding nuclear
Contig8553	46 kda fk506-binding nuclear
Contig8318	78 kda glucose-regulated protein
Contig10913	abcc3 protein
Contig172	actc_biogl ame: full= cytoplasmic flags: precursor
Contig8968	actc_biogl ame: full= cytoplasmic flags: precursor
Contig6084	actin 1
Contig8125	actin-like 6a
Contig8102	acyl-binding protein
Contig11210	acyl-coenzyme a c-2 to c-3 short chain
Contig9591	acyl-coenzyme a long chain
Contig327	acyl-protein thioesterase 1-like
Contig8132	acyl-protein thioesterase 1-like
Contig1128	adenylyltransferase and sulfurtransferase mocs3
Contig496	ADP-ribosylation factor 1
Contig7974	ADP-ribosylation factor 4
Contig10667	ADP-ribosylation factor 6
Contig1407	ADP-ribosylation factor gtpase-activating
Contig10669	ADP-ribosylation factor gtpase-activating protein 1
Contig2449	ADP-ribosylation factor-like protein 2
Contig5180	ADP-ribosylation factor-like protein 3-like
Contig292	aldehyde mitochondrial-like isoform 1
Contig5822	alpha 2 isoform 2
Contig9090	alpha 2 isoform 2
Contig561	alpha tubulin
Contig8359	alpha tubulin
Contig8465	alpha tubulin
Contig6891	alpha-centractin
Contig6203	amino acid
Contig1300	an1-type zinc finger protein 1
Contig7927	angio-associated migratory cell
Contig10296	ankyrin repeat domain-containing protein 50- partial
Contig8034	ankyrin unc44
Contig2952	ARP1 actin-related protein 1 homolog centractin alpha
Contig1993	ARP2 actin-related protein 2 homolog
Contig9106	ARP3 actin-related protein 3 homolog
Contig2412	aryl hydrocarbone receptor nuclear translocator

Contig214	aspartate cytoplasmic
Contig9545	aspartate mitochondrial precursor
Contig8027	aspartate mitochondrial-like
Contig464	ATP synthase subunit mitochondrial-like
Contig5951	ATPase family aaa domain-containing protein 1
Contig8333	ATP-binding cassette sub-family b member mitochondrial precursor
Contig4000	ATP-binding sub-family c (cftr mrp) member 1
Contig7931	ATP-binding sub-family member 5
Contig2090	ATP-dependent metalloprotease, putative [Pediculus humanus corporis]
Contig9406	ATP-dependent RNA
Contig1013	ATP-dependent RNA helicase abstrakt-like
Contig10954	ATP-dependent RNA helicase ddx1
Contig8485	ATP-dependent RNA helicase ddx18
Contig5396	ATP-dependent RNA helicase ddx24
Contig733	ATP-dependent RNA helicase ddx5
Contig8265	ATP-dependent RNA helicase ddx54 isoform 2
Contig9820	ATP-dependent RNA helicase ddx55
Contig2724	ATP-dependent RNA helicase dhx8
Contig707	ATP-dependent RNA helicase me31b-like isoform 1
Contig458	beta 2c
Contig8284	beta 2c
Contig8947	beta 2c
Contig554	beta tubulin
Contig7304	beta-catenin-like repeats containing
Contig9455	bromodomain-containing protein 8-like
Contig4943	calcineurin a
Contig4718	calcium calmodulin-dependent protein kinase type iv-like
Contig341	calumenin isoform 1
Contig9608	casein kinase alpha 1
Contig8058	casein kinase delta
Contig8155	casein kinase ii subunit alpha
Contig2244	cbl-interacting protein kinase 15
Contig8887	cd63 antigen
Contig8983	CDC2 kinase
Contig2342	CDC5l protein
Contig882	cell division cycle 42
Contig10161	cell division cycle protein 20 homolog
Contig8397	cell division protein kinase 10
Contig7863	chaperonin containing subunit 5
Contig9368	chaperonin containing subunit 8
Contig10849	chaperonin containing t-complex polypeptide 1 beta subunit
Contig8161	chaperonin subunit 3
Contig9623	claudin 12
Contig9737	cleavage stimulation 3 pre- subunit 64kda
Contig8021	cleavage stimulation factor subunit 1-like
Contig1426	cmp-sialic acid transporter
Contig4710	coatomer subunit alpha
Contig2108	cop9 signalosome complex subunit
Contig2958	cre-act-2 protein
Contig8952	cre-ubq-1 protein
Contig8904	cullin 1
Contig1211	cullin 4a
Contig7865	cyclin b
Contig8446	cyclin b3
Contig2609	cyclin-dependent kinase 5-like
Contig2470	cyclin-dependent kinase 7-like
Contig5163	cyclin-dependent kinase 9-like

Contig9015	cyclophilin a
Contig8962	cyclophilin type peptidyl-prolyl cis-trans isomerase
Contig8178	cytochrome b5
Contig3143	cytochrome family subfamily polypeptide 13-like
Contig8841	cytochrome family subfamily polypeptide 4
Contig7174	dead (asp-glu-ala-asp) box polypeptide 18
Contig936	dead (asp-glu-ala-asp) box polypeptide 27
Contig6230	dead (asp-glu-ala-asp) box polypeptide 3
Contig493	dead box ATP-dependent RNA
Contig1084	dehydrogenase reductase (sdr family) member 7b-like
Contig5013	dna mismatch repair protein mlh1
Contig261	dna replication complex gins protein psf2-like
Contig262	dna replication complex gins protein psf2-like
Contig595	dna replication licensing factor mcm2
Contig582	dna replication licensing factor mcm3
Contig8774	dna replication licensing factor mcm4
Contig5390	dna replication licensing factor mcm5
Contig7195	dna replication licensing factor mcm6
Contig3745	dnaj homolog subfamily a member 2
Contig287	dnaj homolog subfamily b member 5
Contig7947	dnaj homolog subfamily c member 2
Contig3283	dnaj homolog subfamily c member 5-like
Contig557	dual specificity mitogen-activated protein kinase kinase 5
Contig272	dual specificity protein phosphatase 19- partial
Contig4230	e3 sumo-protein ligase pias3
Contig9575	elav 2-like protein
Contig8080	electron transfer flavoprotein subunit mitochondrial precursor
Contig8940	elongation factor 1 alpha
Contig1475	enoyl- mitochondrial precursor
Contig579	ergic and golgi 2
Contig5786	erk1 2
Contig2294	estrogen receptor
Contig452	eukaryotic initiation factor 4a
Contig8075	eukaryotic translation elongation factor
Contig478	eukaryotic translation initiation factor 2 subunit 3
Contig517	eukaryotic translation initiation factor 3 subunit d-like
Contig9016	eukaryotic translation initiation factor 3 subunit i
Contig8222	eukaryotic translation initiation factor 4e
Contig222	eukaryotic translation initiation factor 4e type 2-like
Contig1618	exonuclease nef-
Contig10995	exosome component 7
Contig727	ezrin radixin moesin
Contig485	f1f0-ATP synthase beta subunit
Contig5129	f-box domain containing protein
Contig4400	f-box wd repeat-containing protein 7
Contig1074	fk506-binding protein
Contig4844	fk506-binding protein
Contig4532	g protein-coupled receptor kinase 5
Contig11185	g1 s-specific cyclin-e1
Contig10358	glucosamine-6-phosphate deaminase 1
Contig4524	glucosamine-6-phosphate isomerase 2-like
Contig9403	glucose regulated protein 75
Contig8079	glutamate-rich wd repeat containing 1
Contig8740	grpe protein homolog mitochondrial-like
Contig4931	gtp binding protein 4
Contig2124	gtp-binding nuclear protein
Contig10246	gtp-binding nuclear protein

Contig169	gtp-binding nuclear protein ran
Contig2381	gtp-binding protein rheb homolog
Contig977	guanine nucleotide-binding protein subunit beta-2-like 1
Contig10353	h aca ribonucleoprotein complex subunit 2-like
Contig2304	h aca ribonucleoprotein complex subunit 3-like
Contig9822	heat shock
Contig453	heat shock 70 kda protein cognate 4
Contig4566	heat shock 70 kda protein cognate 5
Contig891	heat shock protein
Contig580	heat shock protein 60
Contig5215	heat shock protein 70
Contig5202	heat shock protein 70kda
Contig9179	heat shock protein 90
Contig9634	heavy metal tolerance factor 1
Contig177	heterogeneous nuclear ribonucleoprotein 27c-like
Contig8528	heterogeneous nuclear ribonucleoprotein 27c-like
Contig229	heterogeneous nuclear ribonucleoprotein d (au-rich element RNA binding
Contig230	heterogeneous nuclear ribonucleoprotein d (au-rich element RNA binding
Contig889	histone acetyltransferase kat2b
Contig8200	histone-like
Contig3855	histone-lysine n-methyltransferase
Contig8998	hla-b associated transcript 1
Contig8329	homolog 1
Contig8271	homolog subfamily c member 17
Contig8520	hsp90 co-chaperone CDC37-like
Contig10045	hydroxysteroid (17-beta) dehydrogenase 8
Contig8098	hypothetical protein BRAFLDRAFT_280239 [Branchiostoma floridae]
Contig5062	importin subunit alpha-2
Contig5650	importin subunit alpha-7
Contig268	isocitrate dehydrogenase
Contig269	isocitrate dehydrogenase
Contig653	isoform a
Contig8542	isoform a
Contig1182	isoform cra_c
Contig8667	kelch-like protein 8
Contig4308	kidney mitochondrial carrier protein 1-like
Contig4980	kinesin-like protein kif15
Contig4424	kinesin-like protein kif3a-like
Contig1876	lateral signaling target protein 2 homolog
Contig6169	lethal isoform b
Contig6139	leucine-rich repeat-containing protein 47
Contig1331	leucine-rich repeat-containing protein 47
Contig1387	lim domain binding protein
Contig8753	lim homeobox protein cofactor
Contig605	loc394977 protein
Contig435	loc398558 protein
Contig8579	loc495278 protein
Contig4380	loc560667 protein
Contig9500	low quality protein: an1-type zinc finger and ubiquitin domain-containing [illegible]
Contig8224	low quality protein: paraplegin
Contig5592	low quality protein: zinc finger protein partial
Contig5070	lrrc58 protein
Contig6504	lysine-specific demethylase 7
Contig7894	malate dehydrogenase
Contig2044	map kinase kinase 4-like protein
Contig8039	map kinase-activated protein
Contig830	maternal dna replication licensing factor mcm3

Contig3010	maternal embryonic leucine zipper kinase
Contig9113	mcm6-prov protein
Contig3639	member of ras oncogene family
Contig3396	member ras oncogene family
Contig1866	mgc81978 protein
Contig223	mgc89871 protein
Contig6722	microtubule-associated protein rp eb family member 3
Contig155	microtubule-associated rp eb member 1
Contig8826	minichromosome maintenance 7
Contig7929	mitochondrial glutamate carrier protein
Contig2819	mitochondrial solute carrier family 25 member 19
Contig6148	mitogen activated protein kinase kinase isoform cra_b
Contig2550	mitogen-activated protein kinase 14 isoform 2
Contig6875	mitogen-activated protein kinase kinase kinase kinase 5-like
Contig3548	mitogen-activated protein kinase organizer 1
Contig10314	mothers against decapentaplegic homolog 5-like isoform 2
Contig2100	myotubularin-related protein 2
Contig11212	---NA---
Contig8993	nadh dehydrogenase
Contig4688	n-alpha-acetyltransferase catalytic subunit-like
Contig8636	nedd8 precursor
Contig9702	nedd8-activating enzyme e1 catalytic subunit
Contig5423	neuronal spastin
Contig8570	neutral and basic amino acid transport protein rbat
Contig8958	nhp2-like protein 1-like
Contig4012	non-muscle actin
Contig9893	nonmuscle myosin ii
Contig8232	notchless homolog 1
Contig3089	novel protein (zgc:110727)
Contig2105	novel protein vertebrate member of ras oncogene family-like 2b
Contig8644	nuclear receptor 2
Contig524	nucleolar gtp-binding protein 2-like
Contig568	nucleolar protein 56-like
Contig501	nucleolar protein nop52 variant
Contig821	nucleolysin tiar
Contig119	nucleosome assembly protein 1-like 1
Contig7908	nucleosome assembly protein 1-like 1
Contig3589	nucleosome-remodeling factor subunit nurf301-like isoform 1
Contig309	nucleostemin-like protein
Contig10133	oxysterol-binding protein 1
Contig3120	p38 mapk
Contig710	peptidyl prolyl cis-trans isomerase b
Contig1326	peptidyl-prolyl cis-trans isomerase e
Contig511	peptidyl-prolyl cis-trans isomerase fkbp2-like isoform 1
Contig8084	peptidyl-prolyl cis-trans isomerase fkbp4
Contig1798	peptidyl-prolyl cis-trans isomerase h
Contig9214	peptidyl-prolyl cis-trans isomerase-like 1
Contig2456	peptidyl-prolyl cis-trans isomerase-like 2-like
Contig4548	peptidylprolyl isomerase domain and wd repeat-containing protein 1
Contig10273	peptidylprolyl isomerase -like 3
Contig9290	periodic tryptophan protein 2 homolog
Contig972	peroxiredoxin 2
Contig9253	peroxiredoxin 4 precursor
Contig1280	peroxisomal trans-2-enoyl- reductase
Contig1248	phosphatidylinositol 4- beta isoform cra_a
Contig1815	phospholipase a-2-activating protein
Contig8625	pitslre protein kinase alpha sv9 isoform

Contig2900	polycomb protein scm
Contig7877	pre-mrna-processing ATP-dependent RNA helicase prp5
Contig9173	pre-mrna-splicing factor ATP-dependent RNA helicase dhx15-like
Contig9439	pre-mrna-splicing factor ATP-dependent RNA helicase dhx15-like
Contig3025	probable ATP-dependent RNA helicase ddx31
Contig9044	probable ATP-dependent RNA helicase ddx47-like
Contig2072	probable ATP-dependent RNA helicase ddx52-like
Contig8617	probable ATP-dependent RNA helicase ddx52-like
Contig11019	probable ATP-dependent RNA helicase ddx56
Contig1840	probable palmitoyltransferase zdhhc1-like
Contig8048	prohibitin
Contig8982	prohibitin
Contig884	proteasomal ATPase-associated factor 1
Contig7872	proteasome (macropain) beta 6
Contig8618	proteasome (macropain) 26s ATPase 2
Contig2929	proteasome (macropain) 26s non- 10
Contig7913	proteasome (macropain) 26s non- 11
Contig8386	proteasome (macropain) alpha 4
Contig10912	proteasome (macropain) alpha 5
Contig8116	proteasome (macropain) beta 2
Contig8191	proteasome alpha 1 subunit
Contig9053	proteasome alpha 6 subunit
Contig1077	proteasome subunit alpha type-2
Contig8186	proteasome subunit alpha type-3
Contig1781	proteasome subunit alpha type-7
Contig647	proteasome subunit beta type-7
Contig457	protein disulfide-isomerase a3-like
Contig3514	protein disulfide-isomerase a5
Contig1950	protein homolog
Contig2289	protein kinase 3
Contig4072	protein mo25
Contig11059	protein phosphatase 1j
Contig1941	protein phosphatase catalytic subunit
Contig175	protein tumorous imaginal mitochondrial-like
Contig176	protein tumorous imaginal mitochondrial-like
Contig9357	protein tyrosine non-receptor type 2
Contig4469	protein yippee-like 5-like
Contig9765	rad54 homolog b
Contig19	ran binding protein 1
Contig5295	ras protein
Contig2501	ras-related c3 botulinum toxin substrate 1
Contig1717	ras-related c3 botulinum toxin substrate 1 (rho small gtp binding protein r
Contig3148	ras-related protein rab-10
Contig5068	ras-related protein rab-11a-like
Contig11218	ras-related protein rab-14-like
Contig4429	ras-related protein rab-1a
Contig4300	ras-related protein rab-5c
Contig5403	ras-related protein rab-7l1-like isoform 1
Contig6508	replication factor c (activator 1) 37kda
Contig7879	replication factor c subunit 2
Contig8869	replication factor c subunit 3
Contig10185	replication factor c subunit 4
Contig744	replication factor c subunit 5
Contig1237	retinoblastoma binding protein 4
Contig4420	ribose-phosphate pyrophosphokinase
Contig2341	ribosomal protein l14
Contig8999	ribosomal protein l35

Contig240	ribosomal protein s3a
Contig5256	ribosomal protein s6 kinase alpha-
Contig2067	ribosomal protein s6 kinase beta-1-like
Contig9819	ribosomal protein s6 kinase delta-1 isoform 2
Contig9130	ribosomal protein ubq l40e
Contig521	ribosomal RNA methyltransferase nop2
Contig702	ribosome biogenesis protein wdr12 homolog
Contig1080	ribosome production factor 1
Contig9766	rna-binding motif x-linked 2
Contig519	s-adenosylhomocysteine hydrolase
Contig11029	scf complex protein cul-1
Contig8295	septin 6
Contig3686	septin-1-like isoform 2
Contig946	serine arginine-rich splicing factor 2-like
Contig1268	serine threonine protein
Contig998	serine threonine protein
Contig2587	serine threonine-protein kinase chk2-like
Contig6106	serine threonine-protein kinase dclk1
Contig765	serine threonine-protein kinase nek2
Contig8691	serine threonine-protein kinase plk4
Contig499	serine threonine-protein kinase vrk1
Contig1142	serine threonine-protein phosphatase 2a 56 kda regulatory subunit delta
Contig1272	serine threonine-protein phosphatase 2a catalytic subunit alpha isoform-l
Contig11217	serine threonine-protein phosphatase 4 catalytic subunit
Contig1012	serine-arginine repressor protein (35 kda)
Contig9994	serine-threonine kinase receptor-associated protein
Contig1948	serine-threonine kinase-like protein
Contig1692	serum glucocorticoid regulated kinase 1
Contig9315	sfrs protein kinase 2
Contig3986	short chain alcohol
Contig7973	short-chain dehydrogenase
Contig3533	smarcd1 protein
Contig1069	solute carrier family 25 member 42-like
Contig1790	solute carrier family member 27
Contig1356	spastin
Contig1970	spermatogenesis associated 5-like 1
Contig9715	spermatogenesis-associated protein 5-like
Contig3826	steroid receptor-interacting snf2 domain protein
Contig8302	subfamily a member 2
Contig416	subfamily member 1
Contig2419	subfamily member 12
Contig607	sumo1 activating enzyme subunit 1
Contig2744	swi snf-related matrix-associated actin-dependent regulator of chromatin
Contig789	synapse-associated protein
Contig1229	synaptic vesicle-associated gtp-binding
Contig696	taf5-like RNA polymerase p300 cbp-associated factor -associated 65kda
Contig1661	tata box-binding protein 1
Contig734	tata-box-binding protein
Contig6679	t-complex protein 1 subunit
Contig532	t-complex protein 1 subunit alpha
Contig9262	t-complex protein 1 subunit delta
Contig211	t-complex protein 1 subunit theta
Contig7899	t-complex protein 1 subunit theta
Contig1821	t-complex protein 1 subunit zeta
Contig9265	t-complex protein 1 subunit zeta-2-like isoform 1
Contig1434	tfii-f-interacting ctd including nli-interacting
Contig1607	thioredoxin 2

Contig10658	thioredoxin domain containing 9
Contig1810	thioredoxin domain-containing protein c
Contig3457	thioredoxin-dependent peroxide mitochondrial
Contig10392	tpa_inf: myotrophin
Contig691	transcription initiation factor iib
Contig3912	transcriptional repressor protein yy1-like
Contig1011	transducin -like 3
Contig7956	transducin -like 3
Contig1713	transforming protein
Contig563	translation elongation
Contig3617	translation elongation factor tu
Contig413	translocase of outer mitochondrial membrane 70 homolog a
Contig626	triosephosphate isomerase
Contig514	trna (cytosine-5-)-methyltransferase nsun2
Contig428	tubulin alpha-1 chain
Contig503	tubulin alpha-1 chain
Contig10750	tubulin alpha-1 chain
Contig9844	tyrosine kinase receptor cad96ca-like
Contig263	u3 small nucleolar ribonucleoprotein protein imp4
Contig5469	u3 small nucleolar RNA-interacting protein 2
Contig8238	u4 u6 small nuclear ribonucleoprotein prp31
Contig286	u4 u6 small nuclear ribonucleoprotein prp4
Contig7914	u5 small nuclear ribonucleoprotein 40 kda protein
Contig3288	ubiquitin protein
Contig1980	ubiquitin protein ligase e3 component n-recognin 7
Contig419	ubiquitin thioesterase otub1-like
Contig9320	ubiquitin-conjugating enzyme
Contig9061	ubiquitin-conjugating enzyme
Contig1398	ubiquitin-conjugating enzyme
Contig541	ubiquitin-conjugating enzyme e2
Contig3295	ubiquitin-conjugating enzyme e2 11
Contig1531	ubiquitin-conjugating enzyme e2 7
Contig3125	ubiquitin-conjugating enzyme e2 g2
Contig107	ubiquitin-conjugating enzyme e2 q1
Contig9665	ubiquitin-conjugating enzyme e2 q1
Contig7852	ubiquitin-conjugating enzyme e2 s-like
Contig84	ubiquitin-conjugating enzyme e2 s-like
Contig2406	ubiquitin-conjugating enzyme e2c
Contig8994	ubiquitin-conjugating enzyme e2d 3 (ubc4 5 yeast) isoform cra_a
Contig9312	ubiquitin-conjugating enzyme e2n
Contig2231	ubiquitin-conjugating enzyme e2r
Contig4241	ubiquitin-protein ligase e3c
Contig441	uncharacterized protein c19orf29-like
Contig10953	universal minicircle sequence binding protein
Contig954	upf0534 protein c4orf43 homolog
Contig11213	vacuolar h
Contig717	vasa-like protein
Contig463	v-mos moloney murine sarcoma viral oncogene homolog
Contig1907	von hippel-lindau binding protein 1
Contig844	wd domain-containing protein
Contig803	wd repeat protein bub3-like
Contig10012	wd repeat-containing protein 3
Contig228	wd repeat-containing protein 61
Contig825	wd40 repeat-containing protein smu1
Contig4410	yeats domain containing 4
Contig154	zgc:172187 protein
Contig9158	zgc:172187 protein

Contig6657	zinc and double phd fingers family 2
Contig3114	zinc finger
Contig687	zinc finger protein
Contig1608	zinc finger protein 271-like
Contig1139	zinc finger protein 271-like
Contig4087	zinc finger protein 271-like
Contig5280	zinc finger protein 271-like
Contig6183	zinc finger protein 271-like
Contig9147	zinc finger protein 271-like
Contig10996	zinc finger protein 271-like
Contig10807	zinc finger protein 271-like
Contig1739	zinc finger protein 271-like
Contig7054	zinc finger protein 271-like
Contig7960	zinc finger protein 28
Contig2315	zinc finger protein 333-like
Contig4998	zinc finger protein 347
Contig2003	zinc finger protein 429-like
Contig1804	zinc finger protein 568
Contig1965	zinc finger protein 630
Contig6545	zinc finger protein 64 isoforms 1 and 2-like
Contig9383	zinc finger protein 716
Contig3847	zinc finger protein 724-like
Contig4838	zinc finger protein 729- partial
Contig10906	zinc finger protein 845
Contig9736	zinc finger protein 91-like
Contig10452	zinc finger protein 92 isoform 2
Contig4535	zinc finger protein 94
Contig3857	zinc finger protein with krab and scan domains 4
Contig1114	zinc finger protein xfin-like
Contig7950	zinc transporter slc39a7 precursor

In situ validation?	Length	Num	Min E value	Similarity	Human housekeeping blast hit
	374	10	5.20E-20	88%	NM_003404
	412	10	1.00E-34	71%	NM_003404
	822	10	2.90E-104	91%	NM_006761
yes	1631	10	1.60E-83	85%	NM_006761
	1379	10	0.00E+00	93%	NM_006503
	437	10	5.70E-64	91%	NM_002804
	492	10	1.10E-70	94%	NM_002804
	1549	10	0.00E+00	94%	NM_006503
	623	10	4.80E-93	94%	NM_002803
	1526	10	2.30E-179	95%	NM_002805
	1739	10	0.00E+00	96%	NM_002806
	1578	10	3.40E-75	72%	NM_015956
	717	10	5.30E-48	61%	NM_004586
	420	10	2.80E-42	77%	NM_005163
	861	10	2.80E-52	95%	NM_021009
	1893	10	7.20E-38	63%	
	1917	10	7.30E-38	63%	
yes	1062	10	9.00E-105	84%	NM_005347
	374	10	1.20E-32	83%	NM_012089
	1681	10	0.00E+00	99%	NM_005735
	1683	10	0.00E+00	99%	NM_005735
	332	10	2.40E-41	96%	NM_005735
	1561	10	6.10E-166	78%	NM_005735
	866	10	1.50E-26	74%	NM_006117
	1707	10	6.20E-153	87%	NM_014049
	2222	10	9.30E-165	86%	NM_000018
	716	10	4.30E-52	69%	
	678	10	6.20E-42	61%	
	1032	10	9.40E-104	77%	NM_005499
yes	1895	10	3.10E-87	97%	NM_001658
yes	1885	10	1.90E-83	93%	NM_001662
	494	10	3.30E-35	100%	NM_001663
	910	10	7.80E-72	64%	NM_032389
	473	10	3.70E-48	86%	NM_014570
	636	10	5.90E-33	59%	NM_001663
	397	10	1.90E-36	97%	NM_001177
	1516	10	1.30E-176	78%	NM_000696
	356	10	2.90E-47	99%	NM_032704
	2505	10	0.00E+00	99%	NM_032704
	1633	10	0.00E+00	98%	NM_006082
	1076	10	7.70E-136	88%	NM_006082
	1704	10	0.00E+00	99%	NM_032704
	256	10	1.90E-30	98%	NM_005736
	323	10	1.50E-22	75%	
	1056	10	6.50E-47	59%	NM_024699
	1301	10	1.50E-94	66%	NM_001087
	716	10	1.70E-35	56%	NM_032217
	1256	10	1.40E-33	58%	NM_001195098
	574	10	1.30E-75	93%	NM_005736
	754	10	2.70E-95	94%	NM_005735
	2147	10	0.00E+00	93%	NM_005736
	644	10	4.50E-68	80%	NM_001668

	1242	10	9.20E-43	75%	
	796	10	1.90E-84	82%	
	1504	10	1.70E-36	93%	
	2093	10	0.00E+00	89%	NM_004046
	346	10	2.60E-43	91%	NM_032810
	705	10	1.80E-87	83%	NM_012089
	474	10	6.90E-53	81%	NM_012089
	1125	10	1.40E-39	80%	NM_004299
	713	10	1.20E-81	82%	NM_014263
	727	10	4.50E-63	83%	NM_007242
	1101	10	8.20E-152	90%	NM_016222
	358	10	3.00E-37	86%	NM_004939
	979	10	6.20E-126	84%	NM_006773
	384	10	6.40E-31	85%	NM_020414
	1909	10	2.60E-111	92%	NM_006386
	930	10	7.00E-66	92%	NM_024072
	2052	10	1.80E-151	80%	NM_006773
yes	601	10	1.60E-58	96%	NM_004941
	1393	10	0.00E+00	90%	NM_014740
	2152	10	0.00E+00	99%	NM_178014
	1717	10	0.00E+00	100%	NM_178014
	1917	10	0.00E+00	99%	NM_178014
	1657	10	0.00E+00	97%	NM_178014
	201	10	9.70E-19	79%	NM_002264
	860	10	6.20E-44	84%	NM_013263
	479	10	2.50E-83	95%	NM_001009552
	527	10	2.60E-47	74%	NM_006742
	552	10	1.10E-33	67%	NM_001219
	745	10	1.40E-101	96%	NM_001892
	2178	10	2.60E-135	73%	NM_001893
	1203	10	8.00E-148	92%	NM_001256686
	678	10	1.30E-15	54%	NM_004586
	606	10	2.50E-27	67%	NM_001780
	1596	10	6.00E-144	88%	NM_006201
	692	10	7.10E-73	94%	NM_001253
	1209	10	2.10E-92	98%	NM_001791
	2334	10	1.70E-125	61%	NM_016263
	1314	10	1.90E-145	87%	NM_015083
	2082	10	0.00E+00	91%	NM_012073
	705	10	1.00E-90	86%	NM_006585
	325	10	1.70E-31	89%	NM_006430
	1569	10	1.90E-118	86%	NM_005998
	860	10	9.00E-49	77%	NM_033107
	965	10	2.30E-80	76%	NM_015235
	1570	10	6.10E-166	77%	NM_001324
	898	10	4.30E-65	68%	
	428	10	4.70E-67	94%	NM_004371
	708	10	4.30E-119	94%	NM_004236
	756	10	4.60E-127	100%	NM_005735
	441	10	4.60E-67	97%	NM_018955
	1406	10	2.80E-57	86%	NM_003592
	988	10	4.10E-101	84%	NM_003588
	2462	10	1.80E-111	76%	
	2546	10	1.20E-75	68%	
	612	10	7.40E-92	88%	NM_006201
	643	10	9.20E-90	86%	NM_006201
	564	10	9.30E-89	89%	NM_001261

	1720	10	9.10E-61	86%	NM_005729
	1197	10	3.20E-56	82%	NM_005729
	1470	10	1.40E-46	75%	NM_030579
	553	10	3.60E-34	69%	NM_183075
	1969	10	3.10E-54	51%	NM_183075
	637	10	2.60E-68	81%	NM_006773
	1165	10	4.90E-127	82%	NM_017895
	442	10	6.30E-50	94%	NM_001356
	2007	10	0.00E+00	96%	NM_014740
	1102	10	2.10E-72	68%	NM_015510
	410	10	2.70E-53	89%	NM_000249
	986	10	2.00E-63	78%	
	980	10	1.30E-51	77%	
	1554	10	0.00E+00	90%	NM_005916
	1578	10	1.10E-136	87%	NM_005916
	1781	10	0.00E+00	92%	NM_005916
	379	10	1.10E-50	97%	NM_005916
	407	10	6.00E-44	96%	NM_005916
	496	10	8.50E-34	90%	NM_005880
	1432	10	4.90E-54	78%	NM_005880
	1095	10	9.20E-76	73%	NM_014377
	535	10	4.70E-41	88%	NM_025219
	1649	10	5.90E-110	65%	NM_002757
	918	10	5.60E-17	71%	NM_030640
	774	10	3.60E-52	66%	NM_016166
	1862	10	7.20E-168	68%	NM_001419
	1239	10	2.70E-114	84%	NM_000126
	1887	10	0.00E+00	92%	NM_006620
	900	10	1.00E-100	83%	NM_004092
yes	1589	10	3.80E-106	71%	NM_016570
	446	10	6.00E-77	98%	NM_002745
	842	10	1.40E-29	66%	NM_002957
	2213	10	3.30E-178	89%	NM_001416
yes	2934	10	0.00E+00	87%	NM_001961
	1966	10	0.00E+00	93%	NM_003321
	1893	10	0.00E+00	82%	NM_003753
yes	1190	10	2.60E-135	82%	NM_003757
	1793	10	1.20E-100	80%	NM_004846
	1241	10	2.40E-83	78%	NM_004846
	828	10	7.10E-66	71%	NM_020695
	397	10	1.60E-34	75%	NM_015004
	1352	10	1.10E-180	87%	NM_002906
	1931	10	0.00E+00	92%	NM_001686
	403	10	3.60E-45	80%	NM_012300
	502	10	1.40E-65	83%	NM_012300
	1065	10	2.00E-43	85%	NM_002013
	421	10	1.80E-24	82%	NM_002013
	438	10	6.70E-47	88%	NM_003952
	1729	10	5.10E-86	71%	
	470	10	7.60E-39	87%	NM_005471
	442	10	1.20E-32	97%	NM_005471
	623	10	6.60E-77	98%	NM_004134
	1451	10	9.10E-112	68%	NM_031485
	1196	10	2.00E-50	68%	NM_025196
	417	10	7.00E-46	86%	NM_012341
	851	10	5.50E-37	82%	NM_002865
	629	10	1.80E-52	78%	NM_004637

	1774	10	2.20E-97	95%	NM_006325
	656	10	8.10E-78	80%	NM_005402
	1137	10	4.00E-162	90%	NM_019069
	772	10	8.70E-27	72%	NM_017838
	754	10	2.90E-20	85%	NM_018648
	496	10	1.20E-64	96%	NM_005347
yes	2206	10	0.00E+00	95%	NM_006597
	632	10	9.90E-53	71%	NM_004134
	1200	10	4.20E-101	78%	NM_001272049
	1579	10	5.00E-114	91%	NM_001202485
	394	10	4.10E-38	90%	NM_006597
	423	10	2.20E-66	90%	NM_006597
	2607	10	0.00E+00	92%	NM_001272049
	804	10	1.20E-82	88%	NM_004299
	1611	10	2.40E-31	62%	NM_018959
	1723	10	6.20E-49	65%	NM_018959
) protein 37kda)	1195	10	2.10E-44	65%	NM_002138
) protein 37kda)	1105	10	1.90E-44	65%	NM_002138
	1439	10	4.00E-128	80%	NM_003884
	1829	10	6.90E-50	98%	NM_012412
	485	10	6.10E-38	69%	NM_020382
	1496	10	0.00E+00	94%	NM_004640
	1352	10	2.20E-128	84%	NM_012280
	1294	10	5.00E-58	60%	NM_018163
	2221	10	4.00E-89	77%	NM_007065
	565	10	3.40E-30	79%	NM_032783
	898	10	8.80E-20	45%	NM_006565
	408	10	2.40E-41	74%	NM_002264
	363	10	3.60E-34	80%	NM_002264
	1445	10	1.40E-27	82%	
	455	10	6.60E-63	83%	
	1436	10	1.50E-90	64%	NM_012111
	552	10	1.10E-47	78%	
	1005	10	4.90E-81	94%	NM_003345
	623	10	1.90E-65	71%	NM_020803
	456	10	7.60E-40	74%	NM_003562
	411	10	2.90E-43	88%	
	445	10	3.60E-67	96%	
	755	10	4.90E-33	77%	NM_004712
	442	10	3.60E-45	76%	NM_014003
	451	10	4.80E-26	65%	NM_015356
	933	10	9.20E-44	62%	NM_020710
	909	10	2.90E-24	91%	
	1119	10	1.30E-88	89%	
	1534	10	1.20E-157	77%	NM_002669
	2394	10	1.30E-159	80%	NM_015934
	1270	10	3.90E-122	89%	NM_006429
	450	10	1.70E-39	73%	NM_002454
protein 1-like	757	10	2.80E-55	84%	NM_021009
	2041	10	2.20E-108	75%	NM_003119
	370	10	9.10E-22	71%	NM_032584
	403	10	4.20E-33	74%	NM_007373
	294	10	7.00E-33	79%	NM_004459
	1665	10	1.80E-135	83%	
	899	10	3.10E-81	87%	NM_030662
	459	10	6.40E-54	83%	NM_004759
	1250	10	9.80E-157	86%	NM_005916

	554	10	7.30E-62	82%	NM_006251
	1307	10	8.80E-134	78%	NM_005916
	506	10	1.30E-41	80%	NM_002884
	527	10	4.00E-61	95%	NM_021252
	760	10	3.80E-52	61%	NM_003642
	1155	10	2.80E-86	77%	NM_004846
	2912	10	9.10E-78	73%	NM_014268
	2717	10	1.30E-75	72%	NM_014268
	1143	10	1.40E-98	84%	NM_005916
	1222	10	8.70E-102	75%	NM_016016
	584	10	1.90E-34	69%	
	325	10	9.40E-38	82%	NM_002755
	623	10	5.10E-31	79%	NM_002752
	345	10	1.20E-45	86%	NM_004834
	514	10	1.30E-50	84%	NM_019069
	1691	10	8.40E-144	77%	NM_005903
	709	10	4.20E-68	82%	NM_003828
	969	0	-	-	NM_006082
yes	979	10	5.00E-83	84%	NM_002496
	434	10	5.60E-51	96%	NM_003491
	758	10	2.30E-25	97%	NM_006156
	1156	10	7.40E-156	86%	NM_003968
	382	10	2.30E-34	89%	NM_013245
	1047	10	3.70E-51	58%	
	1164	10	1.00E-43	92%	NM_005008
	470	10	2.10E-53	81%	NM_005736
	549	10	1.40E-89	91%	NM_004998
	1825	10	7.10E-175	81%	NM_025234
	1155	10	1.70E-62	81%	
	708	10	4.20E-52	81%	NM_004218
	1258	10	3.40E-52	54%	NM_002957
	1790	10	1.40E-115	61%	NM_013285
	1680	10	1.80E-154	82%	NM_006392
	1869	10	1.70E-44	65%	NM_003683
	1260	10	1.00E-99	80%	NM_003252
	1506	10	3.50E-90	74%	NM_005969
	1466	10	1.90E-88	72%	NM_005969
	511	10	1.80E-57	76%	NM_004459
	1396	10	9.00E-93	73%	NM_014366
	643	10	6.60E-52	83%	NM_014835
	550	10	8.60E-52	68%	NM_002745
	1380	10	1.70E-68	81%	NM_006347
	937	10	2.30E-120	88%	NM_006112
	1904	10	2.70E-45	91%	NM_002013
	1808	10	4.50E-98	69%	NM_002013
	778	10	3.20E-78	88%	NM_006347
	735	10	9.40E-61	86%	NM_015342
	690	10	2.10E-77	82%	NM_015342
	437	10	1.70E-60	88%	NM_015342
	402	10	2.00E-36	88%	NM_015342
	1602	10	8.80E-147	72%	NM_005049
	1142	10	2.80E-91	85%	NM_005809
	1377	10	7.20E-87	85%	NM_002574
	956	10	2.10E-91	73%	NM_032783
	1163	10	5.50E-110	87%	NM_002651
	775	10	3.20E-86	77%	NM_001031689
	975	10	5.20E-132	84%	NM_001261

	579	10	1.20E-65	79%	NM_031488
	1609	10	2.30E-79	58%	NM_016222
	871	10	4.10E-51	97%	NM_001358
	747	10	1.20E-71	90%	NM_001358
	561	10	6.40E-34	57%	NM_004728
	1577	10	1.10E-177	88%	NM_016355
	728	10	1.60E-73	79%	NM_001416
	843	10	6.30E-50	70%	NM_004818
	1783	10	9.80E-164	79%	NM_019082
	765	10	3.20E-35	53%	NM_015457
	1600	10	2.20E-114	89%	NM_001144831
	1303	10	8.40E-106	89%	NM_002634
	1236	10	4.30E-104	64%	NM_000430
	1151	10	5.40E-74	76%	NM_002798
	669	10	3.00E-41	100%	NM_002803
	736	10	2.20E-54	66%	NM_002814
	2025	10	6.20E-158	86%	NM_002815
	920	10	1.10E-97	87%	NM_002789
	1036	10	7.30E-112	91%	NM_002790
	871	10	1.00E-65	78%	NM_002794
	1009	10	3.40E-106	87%	NM_002786
yes	1376	10	6.80E-103	85%	NM_002791
	1090	10	6.30E-109	89%	NM_002787
	1322	10	4.10E-100	88%	NM_002788
	779	10	1.30E-108	89%	NM_002792
	1443	10	1.80E-115	84%	NM_002799
	2150	10	5.10E-144	68%	NM_018981
	514	10	3.60E-21	66%	
	741	10	5.70E-74	78%	NM_000268
	665	10	6.10E-23	50%	NM_004586
	471	10	2.10E-67	94%	NM_016289
	347	10	7.00E-41	96%	NM_001664
	750	10	4.20E-112	94%	NM_002721
	1617	10	4.60E-123	70%	NM_005147
	1539	10	4.30E-123	70%	NM_005147
	949	10	3.00E-82	71%	NM_002827
	448	10	2.70E-37	94%	NM_001005404
	757	10	4.40E-56	81%	NM_001273
	2343	10	2.80E-45	65%	NM_002882
	387	10	1.10E-43	100%	NM_002884
	629	10	2.50E-72	87%	NM_001791
ac1)	789	10	1.10E-90	97%	NM_001791
	552	10	7.40E-59	98%	NM_016131
	406	10	5.10E-60	96%	NM_004218
	1098	10	3.50E-101	97%	NM_016322
	445	10	7.20E-54	95%	NM_004161
	457	10	4.60E-21	59%	NM_004583
	384	10	2.50E-22	70%	NM_016131
	295	10	6.10E-37	86%	NM_007370
	1920	10	1.50E-152	90%	NM_002914
	673	10	4.20E-83	89%	NM_007370
	550	10	3.20E-33	74%	NM_007370
	1335	10	8.70E-146	89%	NM_007370
	976	10	1.80E-148	95%	NM_005610
	447	10	2.50E-51	80%	NM_002766
	679	10	1.50E-48	79%	NM_003973
	631	10	3.80E-37	88%	NM_007209

	1248	10	4.00E-118	90%	
	401	10	1.30E-42	77%	NM_004586
	715	10	2.90E-117	89%	NM_003161
	799	10	4.40E-30	79%	NM_004586
	735	10	6.20E-65	98%	NM_003333
	1792	10	1.40E-157	77%	NM_006170
	1423	10	1.10E-114	65%	NM_018256
	1063	10	2.80E-88	75%	NM_025065
	1288	10	5.10E-55	82%	NM_015235
	1800	10	0.00E+00	90%	NM_000687
	404	10	1.30E-60	94%	NM_003592
	2841	10	1.40E-59	85%	
	497	10	2.10E-66	90%	
	1154	10	1.70E-43	91%	NM_003016
	963	10	1.20E-49	77%	NM_002577
	1115	10	2.80E-115	68%	NM_002710
	621	10	3.90E-59	72%	NM_004759
	332	10	2.90E-27	77%	NM_006742
	1308	10	2.30E-47	69%	NM_003157
	1354	10	1.90E-90	72%	NM_003952
yes	1853	10	1.20E-64	69%	NM_006296
isoform-like	1042	10	3.00E-142	89%	NM_006245
ike	958	10	3.30E-161	98%	NM_001009552
	1517	10	4.80E-154	94%	NM_002720
	1111	10	3.20E-44	90%	NM_006625
	1067	10	9.40E-107	72%	NM_007178
	743	10	5.10E-92	93%	NM_004834
	805	10	2.30E-111	91%	NM_005163
	787	10	1.90E-50	86%	NM_003992
	471	10	6.30E-18	65%	
	934	10	1.30E-70	74%	NM_020905
	510	10	1.30E-52	82%	
	1218	10	4.40E-61	81%	
	779	10	1.60E-82	78%	NM_003562
	929	10	1.80E-57	82%	NM_013245
	737	10	3.50E-57	67%	NM_024063
	872	10	2.60E-79	80%	NM_007126
	487	10	1.00E-63	84%	NM_001273
	1226	10	2.70E-76	74%	NM_005880
	2948	10	2.50E-139	79%	NM_005880
	650	10	3.10E-49	75%	NM_017626
	1520	10	2.40E-86	69%	NM_005500
subfamily d member 1	590	10	2.70E-52	84%	
	1279	10	9.90E-35	89%	NM_004087
	983	10	1.00E-112	96%	NM_016131
	1378	10	1.10E-47	72%	NM_014409
	832	10	7.10E-58	84%	
	1335	10	1.40E-95	92%	
	348	10	1.30E-42	94%	NM_030752
	1743	10	2.00E-150	89%	NM_030752
	1088	10	3.10E-125	91%	NM_006430
	1363	10	3.30E-116	84%	NM_006585
	1270	10	4.80E-98	86%	NM_006585
	772	10	2.10E-94	85%	NM_001762
	937	10	2.40E-57	78%	NM_001762
	896	10	1.00E-105	87%	NM_005730
	830	10	3.50E-33	82%	NM_012473

895	10	3.20E-56	79%	NM_005783
827	10	7.60E-28	66%	NM_005783
552	10	1.50E-39	80%	NM_005809
741	10	5.70E-34	76%	NM_025235
1399	10	1.40E-122	85%	NM_001514
1799	10	6.90E-75	91%	NM_003403
1108	10	3.50E-71	58%	NM_000430
1336	10	3.00E-100	71%	NM_006784
795	10	6.20E-48	91%	NM_001664
1618	10	1.50E-150	77%	NM_003321
501	10	1.10E-35	79%	NM_003321
4257	10	1.00E-120	62%	NM_014820
1489	10	8.50E-87	80%	NM_000365
1881	10	1.20E-143	70%	NM_017755
2478	10	0.00E+00	98%	NM_006082
1875	10	0.00E+00	99%	NM_032704
353	10	1.30E-10	84%	NM_032704
1208	10	6.30E-28	66%	NM_002227
1135	10	1.00E-117	83%	NM_033416
381	10	7.40E-43	77%	NM_014409
1138	10	2.10E-110	77%	NM_015629
1176	10	2.00E-110	85%	NM_004697
1473	10	9.20E-152	84%	NM_004814
564	10	5.90E-62	95%	NM_003336
731	10	3.40E-71	77%	NM_175748
2860	10	3.50E-64	69%	NM_017670
739	10	2.10E-44	69%	NM_003339
841	10	5.00E-67	89%	NM_003340
925	10	5.60E-41	78%	NM_021988
1733	10	2.10E-68	88%	NM_003339
536	10	8.50E-65	88%	NM_003348
858	10	3.30E-55	86%	NM_017811
555	10	2.40E-81	94%	NM_003343
353	10	4.80E-26	86%	NM_017582
488	10	5.90E-32	71%	NM_017582
1193	10	5.20E-75	82%	NM_003339
1072	10	4.40E-75	82%	NM_003339
654	10	3.70E-41	68%	NM_003337
1965	10	8.00E-64	92%	NM_003340
1757	10	9.00E-80	91%	NM_003348
685	10	2.00E-57	92%	NM_017811
461	10	5.40E-62	85%	NM_014671
2288	10	0.00E+00	78%	
2445	10	9.10E-44	54%	NM_003418
1151	10	6.10E-33	64%	NM_002454
1441	10	0.00E+00	93%	NM_001686
1364	10	1.10E-82	77%	NM_001356
2089	10	1.50E-44	54%	NM_004836
751	10	2.60E-58	79%	NM_003372
1229	10	2.00E-97	77%	NM_006784
1284	10	1.60E-106	89%	NM_004725
778	10	5.30E-49	63%	NM_006784
1193	10	2.30E-124	89%	NM_025234
1256	10	0.00E+00	92%	NM_018225
474	10	2.30E-60	85%	NM_005934
840	10	1.70E-74	100%	NM_021009
806	10	5.10E-82	99%	NM_021009

282	10	2.00E-17	87%	
556	10	1.20E-11	61%	NM_006626
1389	10	6.00E-37	84%	NM_006007
822	10	5.40E-31	72%	NM_001099269
1026	10	9.90E-52	65%	NM_003430
466	10	4.30E-51	73%	NM_003430
388	10	3.90E-12	66%	NM_003430
323	10	8.20E-34	77%	NM_003430
765	10	1.60E-42	55%	NM_003430
200	10	1.40E-12	69%	NM_005612
1057	10	5.80E-36	72%	NM_007152
791	10	4.10E-36	60%	NM_007152
232	10	9.70E-17	67%	NM_007152
2143	10	6.00E-69	58%	NM_006991
667	10	3.40E-53	55%	NM_003421
395	10	2.30E-30	73%	NM_003430
666	10	1.10E-12	68%	NM_001271649
775	10	2.30E-34	61%	NM_001135178
733	10	1.70E-14	63%	NM_007130
387	10	7.90E-13	61%	NM_006626
659	10	1.20E-23	59%	NM_003430
455	10	6.90E-18	55%	NM_003430
456	1	9.40E-06	41%	NM_032584
543	10	1.40E-21	69%	NM_032584
309	10	6.40E-31	71%	NM_018657
326	9	6.10E-16	57%	NM_007152
435	10	1.70E-24	61%	NM_007152
486	10	1.50E-27	56%	NM_003421
1045	10	2.80E-34	63%	NM_003430
1207	10	1.40E-65	80%	NM_006979

Number exons	No. GOs	GO IDs	EC
1	10	C:cytoplasm; P:embryo developm	-
1	22	P:axon guidance; P:negative regu	-
1	2	F:protein domain specific binding;	-
1	11	C:cytoplasm; P:embryo developm	-
2	9	P:mitotic spindle elongation; P:ubi	EC:5.2.1.8
1	8	F:protein binding; P:protein catab(	-
1	7	C:cytoplasm; F:peptidase activity;	-
2	6	C:cytoplasm; F:peptidase activity;	-
1	0	-	-
2	9	C:cytoplasm; F:peptidase activity;	-
1	7	C:cytoplasm; F:peptidase activity;	EC:2.7.11.7
	4	F:structural constituent of ribosom	-
5	14	P:regulation of localization; P:regu	-
2	0	-	-
1	4	C:ribosome; F:structural constitue	-
	2	P:cellular protein metabolic proce:	EC:6.3.2.19
	2	P:cellular protein metabolic proce:	EC:6.3.2.19
1	2	F:ATP binding; C:endoplasmic ret	-
5	8	F:xenobiotic-transporting ATPase	-
2	3	C:cytoskeleton; C:cytoplasm; F:A1	EC:1.1.1.37
2	3	C:cytoskeleton; C:cytoplasm; F:A1	EC:2.7.11.0; EC:2.7.10.0
2	0	-	EC:2.7.11.17
2	17	P:histone H4 acetylation; C:nBAF	EC:3.6.1.3
1	1	P:epidermis development	-
1	4	F:flavin adenine dinucleotide bind	-
5	15	P:long-chain fatty acid catabolic p	-
	1	F:carboxylic ester hydrolase activi	-
	1	F:carboxylic ester hydrolase activi	-
1	9	P:enzyme active site formation via	-
1	14	P:regulation of lipid storage; P:reg	-
1	16	P:positive regulation of growth rat	-
1	3	C:intracellular; F:GTP binding; P:s	-
1	3	F:ARF GTPase activator activity; I	-
1	3	F:ARF GTPase activator activity; I	EC:2.7.6.1
1	8	F:protein binding; C:cytoplasm; P:	-
2	21	P:photoreceptor cell development	EC:3.6.3.44
1	2	F:oxidoreductase activity, acting o	-
2	0	-	-
2	0	-	-
2	12	C:tubulin complex; P:mitotic spind	-
2	8	C:microtubule; P:protein polymeri:	-
2	8	C:microtubule; P:protein polymeri:	EC:6.3.2.19
2	7	P:antigen processing and present	EC:3.1.4.12
	3	C:membrane; F:amino acid transr	-
4	1	F:zinc ion binding	-
1	2	C:cell surface; P:smooth muscle c	-
3	0	-	-
3	0	-	-
2	2	F:ATP binding; C:centrosome	-
2	13	C:Arp2/3 protein complex; P:estal	EC:2.7.11.0; EC:2.7.10.2
2	24	P:positive regulation of dendrite m	-
2	3	P:regulation of cellular process; P	-

	11	P:L-methionine salvage from metl	EC:3.6.3.6
	21	P:oxaloacetate metabolic process	-
	16	P:oxaloacetate metabolic process	-
1	19	F:proton-transporting ATPase acti	-
1	10	P:memory; P:learning; C:postsyna	EC:6.3.2.19
5	7	P:transmembrane transport; C:nu	-
5	19	F:lipid-transporting ATPase activit	-
2	5	P:transmembrane transport; C:int	EC:1.3.1.74
2	8	F:zinc ion binding; P:ATP cataboli	-
1	3	F:nucleic acid binding; F:ATP binc	EC:3.4.25.0
2	4	F:nucleic acid binding; F:ATP binc	-
3	3	F:nucleic acid binding; F:ATP binc	EC:5.2.1.8
1	3	F:RNA binding; F:ATP binding; F:/	-
4	5	C:cytoplasm; C:nucleolus; F:ATP-	-
1	24	P:negative regulation of transcript	EC:5.2.1.8
2	9	F:estrogen receptor binding; F:RN	-
1	3	F:RNA binding; F:ATP binding; F:/	-
1	4	F:ATP-dependent helicase activity	EC:6.3.2.19
1	3	F:nucleic acid binding; F:ATP binc	-
2	16	P:natural killer cell mediated cytot	EC:6.3.2.0
2	16	P:natural killer cell mediated cytot	EC:6.3.2.19
2	16	P:natural killer cell mediated cytot	EC:6.3.2.19
2	8	C:microtubule; P:protein polymeri:	EC:6.3.2.19
1	4	C:cytoplasm; P:protein import intc	-
2	0	-	-
2	1	F:phosphoprotein phosphatase ac	EC:3.6.1.15
1	13	P:cellular macromolecule metabol	-
1	2	F:calcium ion binding; C:endoplas	-
1	17	C:cytosol; F:peptide binding; P:pr	-
1	20	P:positive regulation of protein ph	EC:3.6.1.3
1	0	-	-
5	1	F:transferase activity	-
2	2	C:integral to membrane; C:membı	-
3	8	P:protein phosphorylation; F:RNA	-
3	3	F:chromatin binding; F:DNA bindir	-
1	5	F:GTP binding; C:intracellular; F:s	EC:2.6.1.1
1	6	F:protein binding; P:positive regul	EC:3.1.3.16
1	3	F:RNA polymerase II carboxy-tern	-
1	7	C:nucleolus; P:protein folding; C:c	EC:3.1.4.12
1	10	P:'de novo' posttranslational prote	EC:1.11.1.15
1	7	P:protein folding; C:chaperonin-cc	EC:5.2.1.8
2	6	P:negative regulation of retinal ce	-
2	4	P:GTP catabolic process; F:magn	EC:6.3.2.19
1	10	P:mRNA cleavage; C:cleavage bc	-
1	4	F:molecular_function; P:mRNA pr	-
	3	F:substrate-specific transmembra	-
2	5	F:myosin heavy chain kinase activ	-
2	17	F:signal transducer activity; F:tran	-
2	14	P:striated muscle myosin thick fila	-
1	47	P:negative regulation of ubiquitin-	-
1	17	C:nucleoplasm; P:Notch signaling	-
2	5	C:Cul4A-RING ubiquitin ligase coı	-
	5	P:mitosis; F:protein kinase binding	-
	1	P:regulation of cell cycle	-
3	4	P:protein phosphorylation; F:ATP	-
3	3	F:RNA polymerase II carboxy-tern	-
1	12	P:regulation of histone modificatic	EC:1.3.99.3

3	3	P:protein folding; P:protein peptid	-
3	3	P:protein folding; P:protein peptid	-
1	8	C:endoplasmic reticulum membra	EC:5.3.1.1
1	0	-	EC:6.3.2.19
1	1	F:oxidoreductase activity	EC:6.3.2.19
1	3	F:RNA binding; F:ATP binding; F:/	-
3	3	F:nucleic acid binding; F:ATP binc	EC:3.6.1.15
2	3	F:nucleic acid binding; F:ATP binc	-
1	3	F:nucleic acid binding; F:ATP binc	-
2	5	P:oxidation-reduction process; F:c	-
3	25	P:nuclear-transcribed mRNA poly	-
	4	P:S phase of mitotic cell cycle; C:ı	-
	4	P:S phase of mitotic cell cycle; C:ı	-
4	16	C:chromatin; F:protein binding; P:	-
4	18	P:S phase of mitotic cell cycle; P:(	EC:3.1.3.48
4	18	C:chromatin; C:nucleoplasm; P:ce	-
4	14	P:mitotic cell cycle; C:chromatin; I	EC:1.6.5.3
4	11	P:DNA unwinding involved in DN/	EC:2.3.1.88
1	8	C:cytoplasm; P:protein folding; F:I	-
1	4	F:protein binding; P:response to u	EC:2.7.10.0; EC:2.7.11.0
2	6	C:cytosol; F:histone binding; F:ub	-
2	11	F:ATP-dependent protein binding;	-
1	1	F:transferase activity, transferring	-
1	9	P:protein dephosphorylation; P:in	EC:2.1.1.29
8	0	-	EC:2.7.11.0
1	1	F:nucleic acid binding	-
3	2	F:flavin adenine dinucleotide bind	-
2	7	F:nucleotidyltransferase activity; F	-
1	3	P:metabolic process; F:enoyl-Co/	-
1	2	C:cytoplasm; C:intracellular meml	EC:2.7.11.0
3	4	F:MAP kinase activity; P:MAPK ca	-
4	0	-	EC:2.7.11.22; EC:2.7.11.23
3	4	P:translational initiation; F:ATP-de	EC:1.2.1.0
2	9	P:mitotic spindle elongation; C:cyt	-
2	5	F:translation initiation factor activi	-
1	2	P:translational initiation; F:translat	EC:5.2.1.8
2	3	C:eukaryotic translation initiation f	EC:5.2.1.8
2	11	F:translation initiation factor activi	EC:1.1.1.42
2	7	P:translation; C:cytosol; C:mRNA	EC:1.1.1.42
2	3	F:nucleic acid binding; F:exonucle	-
1	0	-	-
2	15	C:filopodium; P:apical protein loca	EC:2.7.11.0
5	8	C:proton-transporting ATP syntha:	-
3	6	P:small GTPase mediated signal t	-
3	16	P:Notch signaling pathway; P:pos	-
1	5	P:protein folding; C:sarcoplasmic	EC:3.6.3.44
1	3	P:protein folding; P:protein peptid	-
1	14	C:cytosol; P:protein autophospho	EC:3.5.99.6
	4	P:primary metabolic process; P:ce	-
2	7	P:carbohydrate metabolic process	-
2	4	P:carbohydrate metabolic process	-
1	13	F:heat shock protein binding; P:pr	EC:3.1.4.12
1	0	-	-
1	4	P:cellular protein metabolic proce:	-
	17	P:NAD biosynthetic process; P:ne	-
2	6	F:GTP binding; C:endoplasmic ret	-
1	3	F:GTP binding; P:small GTPase n	-

2	7	P:small GTPase mediated signal t	EC:5.2.1.8
1	5	P:small GTPase mediated signal t	-
1	7	C:cytoplasm; P:angiogenesis; P:re	-
1	3	F:snoRNA binding; P:rRNA pseud	-
	3	C:nucleolus; P:rRNA processing; (	-
1	5	C:endoplasmic reticulum lumen; F	-
1	1	F:ATP binding	-
1	0	-	-
1	3	P:cellular response to oxidative st	EC:3.1.1.0
2	4	P:protein refolding; C:cytoplasm; I	-
1	0	-	EC:2.7.11.22
1	0	-	-
1	0	-	EC:2.7.7.0; EC:2.8.1.1
2	12	P:cadmium ion transmembrane tra	-
3	2	F:nucleic acid binding; F:nucleotic	-
3	3	F:nucleic acid binding; F:nucleotic	EC:2.7.4.8
1	3	F:nucleic acid binding; F:nucleotic	-
1	3	F:nucleic acid binding; F:nucleotic	-
2	19	F:histone deacetylase binding; F:h	-
3	4	C:nucleosome; F:DNA binding; P:	EC:3.1.4.12
3	1	F:transferase activity	-
1	14	C:U4 snRNP; F:ATP-dependent p	-
1	5	F:rRNA (uridine-2'-O-)-methyltrans	EC:1.8.4.11
1	1	F:binding	-
1	5	C:cytosol; F:Hsp90 protein binding	-
1	3	F:oxidoreductase activity; P:oxida	-
1	3	F:nucleic acid binding; F:zinc ion I	-
1	3	P:protein import into nucleus; F:bi	-
1	4	C:cytoplasm; P:protein import into	-
	5	F:NAD binding; F:isocitrate dehyd	-
	5	F:NAD binding; F:isocitrate dehyd	-
1	4	F:ATPase activator activity; P:pos	EC:3.1.3.16
	7	C:extracellular space; P:protein fo	-
1	23	C:fibrillar center; P:negative regula	-
1	0	-	-
1	3	C:membrane; C:mitochondrion; P	-
	6	C:cytoplasm; P:microtubule-based	-
	6	P:microtubule-based movement; F	-
3	5	C:cytosol; C:early endosome men	-
1	7	F:ATP-dependent RNA helicase a	-
1	1	F:ligase activity	-
2	2	F:RNA binding; F:phenylalanine-tR	-
	3	F:LIM domain binding; F:transcrip	-
	7	F:transcription cofactor activity; P:	-
2	1	C:catalytic step 2 spliceosome	-
3	0	-	-
2	4	P:protein folding; C:cytoplasm; F:u	EC:5.2.1.8
1	1	F:binding	-
1	1	F:zinc ion binding	-
2	10	P:cellular process; F:nucleoside-tr	EC:1.14.19.1
2	1	F:binding	-
1	0	-	-
2	16	F:histone demethylase activity (H4	-
	5	P:cellular carbohydrate metabolic	-
1	17	C:cytosol; P:JNK cascade; P:prote	-
1	8	P:peptidyl-serine phosphorylation;	-
4	14	P:DNA unwinding involved in DNA	-

1	12	P:protein autophosphorylation; F:|	-
4	5	C:MCM complex; F:nucleotide bin	EC:6.3.2.0
2	6	P:small GTPase mediated signal	-
1	4	F:GTP binding; C:plasma membra	-
5	2	P:cellular process; F:transferase a	-
2	7	P:translation; C:cytosol; C:mRNA	-
2	3	F:microtubule binding; P:biologica	EC:3.6.1.15; EC:2.7.7.7
2	13	P:negative regulation of microtubu	EC:3.6.1.15
4	16	C:chromatin; F:protein binding; P:	EC:3.6.4.3
1	2	P:transport; C:membrane	-
	4	C:integral to membrane; C:membr	-
2	11	F:MAP kinase kinase activity; F:pr	-
1	27	P:angiogenesis; P:vascular endot	EC:2.7.11.0
1	0	-	-
1	3	C:cytoplasm; P:mRNA splicing, vi	EC:1.3.1.74
1	7	P:osteoblast differentiation; P:tran	-
5	8	F:protein tyrosine phosphatase ac	EC:2.7.11.16
	0	-	-
1	11	P:response to oxidative stress; P:	-
2	5	C:cytoplasm; P:N-terminal protein	-
3	0	-	-
4	4	F:ATP binding; F:small protein act	EC:3.6.1.3
3	26	C:microtubule organizing center; I	-
	3	P:carbohydrate metabolic process	-
2	7	C:box C/D snoRNP complex; F:sn	-
2	3	C:cytoskeleton; C:cytoplasm; F:A	-
5	4	F:actin binding; C:myosin comple:	-
3	3	C:nucleolus; P:Notch signaling pa	-
	2	F:DNA primase activity; P:DNA re	-
2	2	F:GTP binding; P:small GTPase n	-
4	1	P:in utero embryonic developmen	EC:5.2.1.8
3	3	P:regulation of exit from mitosis; F	-
3	0	-	EC:5.2.1.8
1	10	P:translational initiation; P:viral tra	-
2	4	F:nucleic acid binding; F:oxidored	-
2	2	P:nucleosome assembly; C:nucle	-
2	2	P:nucleosome assembly; C:nucle	-
2	9	C:nucleolus; P:brain development	-
2	5	C:nucleolus; P:GTP catabolic proc	EC:3.6.1.3
5	9	C:nucleolus; F:phospholipid bindir	EC:3.1.4.11
3	13	P:cellular macromolecule metabol	EC:2.7.11.0
6	3	P:protein folding; P:protein peptid	EC:5.2.1.8
2	9	F:RNA binding; P:protein peptidyl-	-
1	5	P:protein folding; P:protein peptid	EC:3.4.24.0; EC:3.6.4.3
1	19	P:protein complex localization; F:I	-
6	12	P:angiogenesis; C:spliceosomal c	EC:5.2.1.8
1	7	P:protein peptidyl-prolyl isomeriza	-
1	5	P:protein folding; P:mRNA splicing	-
1	4	P:protein folding; P:protein peptid	EC:3.3.1.1
1	4	P:protein folding; P:protein peptid	-
4	1	C:intracellular part	-
1	6	C:cytoplasm; P:hydrogen peroxide	EC:3.6.1.3
1	3	P:oxidation-reduction process; F:a	-
1	2	P:metabolic process; F:oxidoredu	EC:6.3.2.19
2	7	P:phosphatidylinositol-mediated s	-
1	4	P:positive regulation of catalytic a	-
1	12	P:protein phosphorylation; P:apop	EC:1.3.99.13

2	3	P:regulation of transcription, DNA	-
2	5	F:helicase activity; F:nucleic acid	-
1	4	F:ATP-dependent helicase activity	-
1	4	F:ATP-dependent helicase activity	-
2	2	F:organic cyclic compound binding	-
1	3	F:nucleic acid binding; F:ATP binc	-
3	3	F:helicase activity; F:nucleic acid	-
1	0	-	-
1	3	F:nucleic acid binding; F:ATP binc	-
2	3	F:metal ion binding; F:transferase	-
1	1	C:membrane	-
1	27	P:progesterone receptor signaling	-
2	1	F:protein binding	EC:3.6.1.15
	3	F:endopeptidase activity; C:protea	-
1	6	C:cytoplasm; F:peptidase activity;	-
2	1	P:lipid homeostasis	-
1	16	P:protein polyubiquitination; C:nuc	EC:3.6.1.3
3	18	P:protein polyubiquitination; C:nuc	-
2	19	P:protein polyubiquitination; C:nuc	EC:2.7.11.23
1	19	P:protein polyubiquitination; F:enc	-
3	8	P:ubiquitin-dependent protein cata	EC:2.7.11.23; EC:2.7.11.22
1	5	C:cytoplasm; C:proteasome core	-
2	5	C:cytoplasm; C:proteasome core	-
2	5	C:cytoplasm; C:proteasome core	EC:3.1.4.12
1	5	C:cytoplasm; C:proteasome core	-
	5	C:cytoplasm; C:proteasome core	-
1	6	P:cell redox homeostasis; F:electr	EC:3.1.4.12
	6	P:cell redox homeostasis; F:electr	-
1	2	C:intracellular part; F:cytoskeletal	-
5	2	P:protein phosphorylation; F:prote	EC:3.6.3.44
1	0	-	-
1	6	F:GTP binding; C:intracellular; F:s	-
1	5	F:metal ion binding; F:protein seri	-
1	1	F:binding	-
1	1	F:binding	-
1	2	F:phosphoprotein phosphatase ac	-
3	2	P:oxidation-reduction process; F:p	EC:3.6.1.15
1	5	F:DNA translocase activity; F:helic	-
2	2	P:intracellular transport; C:intrace	-
2	6	P:small GTPase mediated signal t	-
1	6	F:GTP binding; C:intracellular; F:s	EC:2.6.1.1; EC:2.6.1.7
1	53	C:extrinsic to plasma membrane;	EC:2.6.1.1; EC:2.6.1.7
1	4	F:GTP binding; C:plasma membra	-
2	3	F:GTP binding; P:small GTPase n	-
2	3	F:GTP binding; P:small GTPase n	-
3	6	F:GTP binding; C:Golgi apparatus	-
2	1	C:cytoplasmic part	-
1	1	F:nucleotide binding	-
1	9	F:nucleoside-triphosphatase activ	-
1	7	F:nucleoside-triphosphatase activ	EC:2.7.11.23
1	8	P:response to organophosphorus	-
1	2	C:DNA replication factor C comple	-
1	6	F:nucleoside-triphosphatase activ	EC:2.7.11.0
2	13	P:mitotic cell cycle; P:CENP-A co	-
1	5	P:nucleotide biosynthetic process	-
	1	C:ribonucleoprotein complex	-
	10	P:translational initiation; P:viral tra	-

	13	P:nuclear-transcribed mRNA cata	-
5	3	P:phosphorylation; F:protein serin	-
2	50	C:cell surface; P:response to elec	-
5	6	F:phosphatidylinositol binding; C:e	-
1	7	C:microtubule associated complex	-
4	5	C:nucleolus; F:S-adenosylmethion	-
2	2	C:nucleolus; P:rRNA processing	-
1	3	C:nucleolus; F:RNA binding; P:rib	-
1	2	F:nucleotide binding; F:RNA bindi	-
2	4	C:cytoplasm; P:S-adenosylmethio	EC:3.6.4.3
1	0	-	EC:3.6.3.6
	3	F:GTP binding; P:cell cycle; C:sep	-
	11	P:smoothened signaling pathway;	-
1	2	F:nucleic acid binding; F:nucleotic	EC:5.2.1.8
2	3	P:phosphorylation; F:protein kinas	-
2	1	F:hydrolase activity	-
1	8	P:regulation of primary metabolic	-
1	4	P:central nervous system develop	-
3	7	P:protein phosphorylation; P:orga	-
1	8	P:protein phosphorylation; P:posit	EC:3.5.99.6
1	5	P:protein autophosphorylation; F:p	EC:2.7.11.0
3	8	P:cellular response to growth fact	-
2	17	P:proteasomal ubiquitin-depender	-
2	1	F:phosphoprotein phosphatase ac	EC:3.1.3.16
2	10	F:RNA binding; P:mRNA 5'-splice	-
1	5	P:RNA processing; C:intracellular	EC:3.4.25.0
1	0	-	-
2	27	F:cofactor binding; P:positive regu	-
1	20	P:angiogenesis; P:positive regulat	-
	2	P:embryo development ending in	-
1	1	P:metabolic process	-
	7	C:npBAF complex; C:nBAF comp	-
	3	C:mitochondrial inner membrane;	-
1	1	C:membrane	-
3	23	C:microtubule organizing center; I	-
1	4	F:ATP binding; F:nucleotide bindir	-
2	6	P:spermatogenesis; P:cell differer	EC:3.4.25.0
1	5	F:ATP binding; F:helicase activity;	-
1	2	F:metal ion binding; F:protein binc	EC:2.7.11.24
1	14	F:heat shock protein binding; P:sp	-
1	1	F:protein binding	-
2	10	F:ubiquitin activating enzyme activ	-
	0	-	-
2	1	F:guanylate kinase activity	EC:2.7.7.0
1	4	F:GTP binding; F:sphingomyelin p	EC:2.7.11.0; EC:2.7.10.0
1	3	F:transferase activity, transferring	EC:3.6.1.15
	5	F:DNA binding; P:transcription init	-
	7	P:gastrulation; P:transcription initi	-
1	4	P:protein folding; C:cytoplasm; F:u	-
1	4	P:protein folding; C:cytoplasm; F:u	-
1	4	P:protein folding; C:cytoplasm; F:u	EC:5.2.1.8
1	5	P:protein folding; C:microtubule o	-
1	5	C:cytoplasm; P:protein folding; P:I	EC:2.7.1.67
1	5	C:chaperonin-containing T-comple	-
1	4	P:protein folding; C:cytoplasm; F:u	-
1	11	P:protein dephosphorylation; P:nu	EC:1.14.11.27; EC:1.13.11.
1	6	C:nucleolus; F:electron carrier act	EC:3.1.4.12

1	1	P:cell redox homeostasis	-
1	0	-	-
1	3	P:oxidation-reduction process; F:a	-
3	0	-	-
2	5	P:translational initiation; P:DNA-d	-
1	3	F:zinc ion binding; F:nucleic acid l	-
2	3	P:lymphocyte differentiation; P:thy	EC:3.6.4.3
1	4	P:lymphocyte differentiation; P:thy	-
1	4	F:GTP binding; C:intracellular; C:p	-
2	4	P:primary metabolic process; F:tra	-
	6	F:translation elongation factor acti	-
1	1	C:mitochondrion	EC:2.7.11.0
1	4	P:gluconeogenesis; F:triose-phos	EC:3.6.1.3
1	4	F:tRNA (cytosine-5-)-methyltransf	-
2	12	C:tubulin complex; P:mitotic spind	-
2	8	C:microtubule; P:protein polymeri	EC:6.3.2.19
	8	C:axonemal microtubule; P:protei	-
5	5	F:ATP binding; F:protein kinase a	-
2	2	F:nucleic acid binding; C:ribonucle	EC:6.3.2.19
1	0	-	-
1	12	C:U4 snRNP; F:snRNP binding; C	-
2	7	C:U4/U6 snRNP; P:RNA splicing;	EC:2.3.1.48; EC:2.3.1.32
1	6	C:cytoplasm; C:nucleolus; P:RNA	-
2	3	P:protein ubiquitination; F:ATP bir	-
1	2	F:metal ion binding; F:ligase activ	EC:6.3.2.0
2	7	P:negative regulation of double-st	-
2	4	F:ATP binding; F:ligase activity; F	-
2	23	P:cyclin catabolic process; P:dete	-
2	1	F:ubiquitin-protein ligase activity	EC:1.11.1.15
2	15	P:cyclin catabolic process; F:prote	-
2	2	F:ubiquitin-protein ligase activity; l	-
1	3	P:protein ubiquitination; F:ATP bir	-
4	3	P:protein ubiquitination; F:ATP bir	-
1	1	F:acid-amino acid ligase activity	EC:2.7.11.0
1	1	F:acid-amino acid ligase activity	-
2	9	P:protein K11-linked ubiquitinatior	-
2	9	P:protein K11-linked ubiquitinatior	-
2	15	P:cyclin catabolic process; P:posi	EC:3.6.1.3
2	20	P:transcription initiation from RNA	EC:3.6.1.3
2	28	P:DNA damage response, detecti	-
1	4	P:protein ubiquitination; F:ATP bir	EC:3.6.4.3
1	3	P:protein polyubiquitination; F:ubi	-
	0	-	-
1	4	F:nucleic acid binding; P:regulatio	-
1	3	F:molecular_function; P:biological	-
5	12	F:proton-transporting ATPase acti	-
2	0	-	-
6	6	F:ATP binding; F:protein kinase a	-
1	3	P:protein folding; C:prefoldin com	-
1	2	C:nuclear membrane; C:nucleolus	-
1	6	P:attachment of spindle microtubu	-
1	3	C:nuclear membrane; C:nucleolus	-
3	10	C:CDC73/Paf1 complex; P:negati	-
2	2	C:nucleus; C:cytoplasm	-
3	2	C:nucleus; P:regulation of transcri	-
1	1	F:molecular_function	-
1	3	C:nucleus; C:cytoplasm; F:protea	-

	3	F:zinc ion binding; F:nucleic acid I	-
1	3	F:nucleic acid binding; F:zinc ion I	-
1	2	F:zinc ion binding; F:DNA binding	-
2	0	-	-
3	0	-	EC:4.2.1.17; EC:4.2.1.74
3	0	-	-
3	0	-	EC:2.7.11.24
3	3	P:transcription, DNA-dependent; I	-
3	0	-	-
1	0	-	EC:3.6.1.15; EC:3.4.24.0
2	0	-	-
2	0	-	EC:3.4.25.0
2	1	F:binding	EC:3.4.25.0
1	7	P:regulation of transcription, DNA	-
2	3	F:nucleic acid binding; F:zinc ion I	-
3	1	P:transcription, DNA-dependent	-
1	1	F:binding	EC:3.1.1.0
1	1	F:binding	-
1	7	P:regulation of transcription, DNA	EC:3.4.25.0
1	3	F:nucleic acid binding; F:zinc ion I	-
3	9	F:metal ion binding; P:regulation c	-
3	0	-	-
2	0	-	-
2	4	F:metal ion binding; P:transcriptio	EC:6.3.2.19
1	0	-	-
2	2	F:binding; C:nucleolus	EC:3.4.25.0
2	1	F:metal ion binding	EC:3.4.25.0
2	5	F:metal ion binding; P:regulation c	EC:2.7.7.0
3	5	F:metal ion binding; P:regulation c	-
1	4	C:membrane; P:zinc ion transport	EC:3.6.1.15
